# Deficiency of GLUT1 in macrophages reprograms tumor-associated macrophages to normalize tumor vasculature and retards the progression of tumors

**DOI:** 10.7150/ijbs.131425

**Published:** 2026-07-30

**Authors:** Yuan Fang, Wei Zou, Ling Ning, Tianle Li, Ying Huang, Xiaoxiong Song, Mengyuan Gao, Ruiqin Jia, Mingxi Zhu, Wenshi Qiu, Kexin Liu, Aiyun Wang, Jia Li, Yin Lu, Yang Zhao

**Affiliations:** 1 Jiangsu Key Laboratory for Pharmacology and Safety Research of Chinese Materia Medica, School of Pharmacy, Nanjing University of Chinese Medicine, Nanjing 210023, China.; 2 Department of Biochemistry and Molecular Biology, School of Medicine, Nanjing University of Chinese Medicine, Nanjing 210023, China.; 3 School of Pharmacy, Henan University, Kaifeng 475001, China.; 4 Curtin Medical Research Institute and Curtin Medical School, Curtin University, Bentley, WA, Australia.; 5 Perron Institute for Neurological and Translational Research, Nedlands, WA 6102, Australia.; 6 State Key Laboratory of Mechanism and Quality of Chinese Medicine, Macau University of Science and Technology, Taipa 999078, Macau SAR, China.

**Keywords:** tumor-associated macrophages, GLUT1, vascular normalization, CD36, nanoparticles

## Abstract

Tumor-associated macrophages (TAMs), a major glucose-consuming population within the tumor microenvironment (TME), utilize glycolysis to support tumor progression. Therefore, concentrating on the glycolysis of intratumoral TAMs appears to be a promising research direction for tumor therapy. In this study, we demonstrated that the key glycolytic component named glucose transporter protein 1 (GLUT1, also known as SLC2A1) is extensively expressed in TAMs and is frequently associated with tumor progression. To dissect the functions of GLUT1 in macrophages on influencing the TME, macrophage-specific *Glut1* knockout mice were generated and tumor-bearing mouse models were established. It was found that macrophage-specific deletion of Glut1 retarded the development of tumors and reshaped the tumor immune microenvironment (TIME). Loss of *Glut1* in macrophages limited the secretion of multiple inflammatory mediators, including IL-6, IL-10, VEGF, and CXCL2, by virtue of the GSK3β/β-catenin/CD36 signaling axis, thereby normalizing the tumor blood vessels. To pharmacologically manipulate the expression of Glut1 in macrophages, M2-PLGA@WZB117 (poly lactic-co-glycolic acid, PLGA) was constructed accordingly. Interestingly, M2-PLGA@WZB117 resulted in significant inhibition in tumor progression, accompanied by tumor vascular normalization and a concurrent increase in the infiltration of CD8^+^ T cells. Therefore, our study provides new insights into reprogramming TAMs to exert impacts on tumor endothelium and thus regulating the TME, thereby hindering the development of tumors.

## Introduction

Cancer is one of the major causes of death around the world, posing significant health challenges to all humanity. The occurrence and development of malignant tumors are complicated and multi-stage biological processes involving crosstalk between different types of cells 1, 2. Tumor-associated macrophages (TAMs) are the innate immune population with the richest infiltration into the tumor microenvironment (TME) 3. It has been widely held that TAMs play important roles in influencing tumor cell proliferation, metastasis, angiogenesis, immunosuppression, extracellular matrix remodeling, and resistance to chemotherapeutic drugs and checkpoint blockade immunotherapy 4-7. These multifaceted interactions underscore the critical contribution of TAMs to the TME, thereby facilitating tumor progression and complicating therapeutic interventions 8. TAMs can be divided into two categories based on classic classification of macrophages: M1-like macrophages that exhibit pro-inflammatory and anti-tumor properties, and M2-like macrophages that exert anti-inflammatory and pro-tumor functions 5. Consequently, elucidating the molecular mechanisms governing TAM polarization and functional modulation is imperative for the development of targeted strategies aimed at reprogramming these macrophages to exert anti-tumorigenic effects, ultimately enhancing the efficacy of existing therapeutic modalities 9, 10.

It has been well documented that cancer cells tend to sustain rapid proliferation and malignant progression primarily through aerobic glycolysis, a phenomenon known as the Warburg effect, which appears to be the pivotal characteristic of tumor metabolic reprogramming 11-14. Of note, recent studies have unveiled that the increased glucose metabolism in the TME predominantly stems from the infiltrating immune cells, in particular TAMs, rather than the tumor cells themselves 15. This challenges the conventional view that tumor cells are the main glucose consumers in the TME and suggests that therapies targeting tumor cell glycolysis alone may be insufficient. Indeed, reprogramming glycolysis in macrophages has gradually gained increasing attention in serving as a promising treatment strategy 16. Recent reviews have highlighted that TAMs are highly glycolytic and that targeting their metabolism can broadly modulate the TME, supporting the rationale for macrophage-focused therapeutic strategies 17. Given that glucose-derived metabolites in TAMs can act as signaling molecules to drive pro-metastatic programs 18, the regulation of glucose uptake becomes a critical focal point. Glucose transporter protein 1 (GLUT1, also referred to as SLC2A1) preferentially facilitates glucose transport into cells and plays a crucial role in influencing cellular energy metabolism. Its expression levels are tightly regulated in response to metabolic demands and are frequently upregulated in multiple pathological conditions, including malignancies, thereby leading to altered metabolic phenotypes associated with disease progression 19, 20. However, whether GLUT1 in macrophages is able to exert any impacts on regulating tumor development remains unclarified.

Although tumor cells are inclined to obtain carcinogenic mutations that enable sustained proliferation and evasion of cell death, they cannot grow in the absence of a vascular system that delivers oxygen and nutrients. In fact, angiogenesis plays a fundamental role in transforming benign tumors to malignant tumors 11. More importantly, it has been increasingly recognized that TAMs exert robust impacts on promoting the formation of tumor vascular networks 21. M1-like macrophages are able to restrict angiogenesis by virtue of boosting the secretion of various antiangiogenic cytokines including interleukin-12 (IL-12) and tumor necrosis factor-alpha (TNF-α) 22, whereas M2-like macrophages augment tumor angiogenesis through releasing multiple angiogenic chemokines (such as CXCL8 and CXCL12) and growth factors (such as VEGF, FGF, EGF and PDGF-b) 21, 23-25. Furthermore, it has been recently illustrated that metabolic competition for glucose between TAMs and tumor-associated endothelial cells (TECs) tends to mitigate the production of aberrant vascular networks 26. Consistently, recent studies have shown that hypoxic TAM niches are able to directly influence vascular function and T cell infiltration, further highlighting the critical role of TAM metabolism in shaping tumor vascular networks 27. As such, all of these findings pinpoint the preponderant role of interactions between TAMs and tumor blood vessels in exacerbating tumor progression.

In the present study, we demonstrated that the mice with macrophage-specific deletion of *Glut1* displayed markedly reduced susceptibility to the growth of colorectal and lung tumors. More specifically, macrophage-specific knockout (KO) of GLUT1 remodeled the TME, as evidenced by the inhibited tumor angiogenesis and enhanced vascular normalization, which was mediated through limited secretion of various inflammatory components. Mechanistically, these effects are ascribed to the modulation of the GSK3β/β-catenin/CD36 signaling axis upon loss of GLUT1 in macrophages. Although previous studies have revealed broad effects of TAM metabolism on vascular function, the precise molecular mechanisms remain unclear. Our study specifically identifies the GLUT1/GSK3β/β-catenin/CD36 signaling axis as this critical mechanistic link, providing a novel perspective on how TAM metabolism shapes the TME. Furthermore, aiming at pharmacologically manipulating the expression of Glut1 in macrophages, M2-PLGA@WZB117 (M2 macrophage-targeted nanoparticles encapsulating the GLUT1 inhibitor WZB117) was constructed accordingly. It was shown that M2-PLGA@WZB117 led to significant repression in tumor progression, accompanied by tumor vascular normalization and an increased infiltration of CD8^+^ T cells. Taken together, our study highlights the potential interactions between TAMs and TECs, and also provides a proof of concept that targeting GLUT1 in macrophages may represent a promising therapeutic strategy for cancer patients.

## Materials and Methods

### Reagents and antibodies

Antibodies against GLUT1 (ab115730) and Collagen IV (ab6586) were obtained from Abcam Technology (Cambridge, USA). Antibody against CD36 (SC-7309) was obtained from Santa Cruz Biotechnology (Santa Cruz, USA). Antibodies against CD68 (A23205), CD8 (A23081), CD86 (A16805), p-GSK3-S9 (ap0039) and Phospho-β-catenin (AP1315) were obtained from Abclonal (Wuhan, China). Antibodies against AKT (4691L), GSK3β (124565), β-catenin (8480s), CD206/MRC1 (24595), Claudin-5 (49564S), VE-cadherin (2500S), α-SMA (19245s) and ZO-1 (13663S) were obtained from Cell Signaling Technology (Danvers, USA). Antibodies against Ki67 (14-5698-82), IFN gamma (MM700), Granzyme B (14-8823-52), F4/80 (14-4801-82), phospho-Akt (Ser473) (RA2102) and TUNEL kit (A11301) were purchased from Vazyme (Nanjing, China). CD31 (557355) and CD31-PE (553373) were obtained from BD Biosciences (San Jose, USA). Antibodies against β-Actin (66009-1-lg) and LaminB1 (66095-1-lg) were from Proteintech (Wuhan, China). Goat Anti-Mouse/Rabbit IgG(H^+^L) HRP (BS12478/BS13278) were obtained from Bioworld (Nanjing, China). Goat anti-Rat IgG (H^+^L)-Alexa Fluor™ 488 (A11006), Goat anti-Rat IgG (H^+^L)-Alexa Fluor™ 594 (A11007), Goat anti-Mouse IgG (H^+^L)-Alexa Fluor™ 488 (A11001) and Goat anti-Mouse IgG (H^+^L)-Alexa Fluor™ 594 (A11005) were acquired from Invitrogen (Carlsbad, USA). CD45-FITC (157608), F4/80-PE/Cyanine7 (123114), CD11b-APC (101211), CD86-PerCP/Cyanine5.5 (141706), CD206-PE (141706), CD3-APC (100236), CD4-FITC (100406) and CD8-PE (100708) were acquired from Biolegend (San Diego, USA).

### Animals and experimental design

*Glut1*^fl/fl^ mice 28 and* Csf1r*^cre/+^ mice 29 (on C57BL/6J background) were purchased from Shanghai Model Organisms Center, Inc. *Glut1*^fl/fl^ mice were crossed with *Csf1r*^cre/+^ mice to obtain *Glut1*^fl/fl^*Csf1r*^cre/+^ mice (as KO group) or *Glut1*^fl/fl^*Csf1r*^+/+^ mice (as control group). The genotype of mice was determined by PCR in combination with agarose gel electrophoresis. Analysis was performed on the mice (6-8 weeks old) obtained from the abovementioned breeding. For the subcutaneous tumor models, 2 ×10^6^ MC38 and LLC cells were subcutaneously injected into the mice.

Furthermore, the C57BL/6J mice (6-8 weeks old) obtained from Jiangsu Huachuang Xinnuo Pharmaceutical Technology Co., Ltd. were used for the experiments of WZB117 and M2-PLGA@WZB117. In brief, 1×10^6^ MC38 cells were subcutaneously injected into the mice. Starting from the eighth day, the mice received intraperitoneal injection of formulations at WZB117 equivalent doses of 5 or 10 mg/kg every two days (the dosage was calculated according to the encapsulated WZB117 payload within M2-PLGA@WZB117 nanoparticles). After six times of treatment, the mice were sacrificed through cervical dislocation, and the tumor tissues were harvested for further analysis.

Tumor size was monitored at regular intervals of 2-3 days by measuring the length and width with a digital caliper. The tumor volume was calculated based on the following formula: tumor volume = (length × width^2^)/2.

### Ethics approval and consent to participate

In accordance with the approval requirements of the Medical Ethics Committee of Red Cross Hospital of Yulin City, Guangxi (Ethics Number: 202501), the primary human lung cancer tissue samples and colon cancer tissue samples were collected in the Red Cross Hospital of Yulin City, Guangxi. Patients' consent forms were obtained from all participants before their inclusion into the study. The collected paraffin sections were used for IF staining.

All animal experiments were performed in compliance with international standards for laboratory animal welfare, with prior approval from Nanjing University of Chinese Medicine Institutional Review Board (Ethical Review Number: 202405A009 and 202506A041).

### Macrophage isolation

TAMs were isolated from the tumor tissues using FACS. Briefly, the tumor tissues were prepared into single-cell suspensions as described above. Subsequently, the cell suspension was blocked with FcR blocking solution for 10 min, and incubated with fluorophore-conjugated antibodies for 30 min. Ultimately, the CD45^+^CD11b^+^F4/80^+^ cells were sorted using the BD FACS Aria^TM^ Fusion Cell Sorter. bone marrow-derived macrophages (BMDMs) were generated as previously described 30. In brief, the BMDMs were obtained via flushing the femurs and tibias of 5-to-6-week-old mice. After erythrocyte lysis with red blood cell lysis buffer for 5 min at room temperature, the cells were washed twice with ice-cold phosphate buffered saline.

### Immunofluorescence (IF) staining

The tissue samples were embedded with optimal cutting temperature (OCT) compound or paraffin, and were subsequently prepared into frozen sections or paraffin sections. The frozen sections were fixed with 4% paraformaldehyde (PFA) and permeabilized with Triton X-100 (0.5%). The sections were then blocked with goat serum blocking solution, incubated with indicated primary antibodies and corresponding secondary antibodies with fluorescent groups respectively, and ultimately stained with Hoechst (BP-DL-711, Senbeijia, Nanjing, China) or DAPI (C1022, Beyotime, Shanghai, China). Furthermore, the paraffin sections were stained according to the instructions of the multiplex fluorescence staining Kit (AFIHC024, Aifang Bio, Hunan, China), which made use of tyramine signal amplification technology. The random fields of sample were imaged under a fluorescent microscope (Leica Thunder, Wetzlar, Germany).

### Hematoxylin-eosin (H&E) staining

The samples were stained by using the H&E Staining Kits according to the manufacturer's protocol. The images were acquired using the Mantra Pathology Workstation (PerkinElmer, Waltham, USA).

### Flow cytometry

The tumors were excised, minced and dissociated in the digestion buffer (2 mg/mL collagenase IV and 1 mg/mL DNase I in RPMI 1640). The tissue samples were incubated for 30 min at 37 °C with agitation and then filtered through 70 μm cell strainer to remove undigested tumor tissues. Following red blood cell lysis, the single cell suspension was resuspended in the flow cytometry staining wash buffer. Subsequently, cells were blocked with FcR blocking solution (C1755S, Beyotime, Shanghai, China) for 10 min on ice and incubated with indicated fluorescent conjugated antibodies for 30 min in the dark. The data were obtained on the Beckman flow cytometer (Gallios or CytoFLEXS) (Miami, USA) and analyzed with FlowJo V10.

### Cell culture

The mouse Lewis lung carcinomas cells (LLC), the mouse colon cancer cells (MC38) and the mouse leukemia cells of monocyte macrophage (RAW 264.7) were obtained from the American Type Culture Collection (ATCC). The cells were grown in the DMEM medium (12800017, Gibco, Grand Island, USA) containing 10% fetal bovine serum (FBS) (F101, Vazyme, Nanjing, China) and 1% penicillin-streptomycin solution at 37 °C in a humidified atmosphere containing 5% CO_2_. Human umbilical vein endothelial cells (HUVECs) were isolated and purified from the donated umbilical cords as previously described with minor modification 31, as approved by the Medical Ethical Committee of Jiangsu Province Hospital on Integration of Chinese and Western Medicine (permit and approval number: 2021-LWKY-003). Written informed consent was obtained. HUVECs were used at low passage numbers between 2 and 5. All the cells used in this study were validated to be free of mycoplasma contamination.

### Conditioned media preparation

To prepare for the tumor-conditioned medium (TCM), the MC38 colon cancer cells were plated in the DMEM medium in dishes. After 24 hours, the culture medium was collected and centrifuged at 2000 rpm for 10 min, filtered by using 0.22 μm filter and then stored at -20 °C. After treating macrophages with TCM for 24 hours, the macrophages turned to be TAMs. To prepare for TAMs-conditioned media (TAMs-CM), following the treatment of TCM, the culture medium was collected according to the steps mentioned above, centrifuged and filtered.

### Lentiviral transduction

Lentivirus (ZsGreen-Puro)/NC and Lentivirus (ZS Green-Puro)/U6-*Glut1* were purchased from Corues Biotechnology (Nanjing, China). The oligo sequences were listed in detail in the Supplementary Table S1.

The RAW264.7 cells were infected with lentivirus diluted in the DMEM medium without FBS and penicillin-streptomycin solution for 24 hours, and subsequently cultured in the fresh complete medium for 72 hours. After incubation with puromycin (6 μg/mL) for 48 hours, the cells were collected and then the transfection efficiency was evaluated by qRT-PCR and Western blot analysis.

### RNA sequencing (RNA-seq)

TAMs were isolated from the tumor tissues of *Glut1*^fl/fl^*Csf1r*^cre/+^ mice and *Glut1*^fl/fl^ *Csf1r*^+/+^ mice. In addition, the RAW264.7 cells were transformed into TAMs using TCM and were divided into the control group and the knockdown group. Two groups of samples were subjected to RNA sample preparation, library preparation, library quality inspection and transcriptome sequencing by Gene Denovo Biotechnology Co. (Guangzhou, China). RNA sequencing analysis was performed using the Omicsmart sequencing platform (https://www.omicsmart.com/).

### Western blot

The total proteins were extracted using the pre-cooled RIPA lysis buffer (P0013C, Beyotime, Shanghai, China). Nuclear and cytoplasmic proteins were obtained using the Nuclear and Cytoplasmic Protein Extraction Kit (P0027, Beyotime, Shanghai, China). The lysates were then separated by SDS-PAGE electrophoresis. After transferring the proteins to the polyvinylidene fluoride (PVDF) membrane, the PVDFs membranes were blocked with bovine serum albumin (BSA), and then incubated with indicated primary antibodies and corresponding HRP-conjugated secondary antibodies. Detection was performed using the enhanced chemiluminescence (ECL) kit, and the images were acquired using the chemical XRS^+^ system (Bio-Rad, Hercules, USA). In order to perform the quantitative analysis, each western blot experiment was repeated at least three times.

### RNA extraction and RT‒qPCR

Total RNA was isolated from the cells by using the FreeZol Reagent (R711-01). According to the manufacturer's instructions, the reverse transcription reagent (R223-01) was employed to reverse transcribe RNA into cDNA. Quantitative reverse transcription PCR (RT-qPCR) was performed with SYBR Green qPCR Master Mix (Q331). All reagents used for the assay were purchased from Vazyme (Nanjing, China) The quantification of mRNA expression was performed via the comparative Ct (2^-ΔΔCt^) method. The primer sequences used in the study were listed in detail in Supplementary Table S2.

### Vascular perfusion and leakage

To determine the vascular perfusion and leakage in tumors, 100 μL LECTIN (1 mg/mL, DyLight488, Vector, Burlingame, USA) or TRITC-dextran (25 mg/mL, 70 kDa, Sigma Aldrich, St. Louis, USA) were intravenously injected into the tumor-bearing mice on the final day of the tumor growth experiment 32. After 60 min of circulation, the tumor tissues were embedded with OCT compound and sectioned. Co-staining for CD31 and DAPI was performed according to the standard IF staining protocol, and the vascular perfusion and vascular leakage in tumors were evaluated accordingly.

### Migration assay

The effects of TAMs-CM on the migration of endothelial cells (ECs) were determined by the wound healing assay. Briefly, the ECs were plated into the 6-well plates and grown to form a confluent monolayer. Subsequently, a sterile 200 µL pipette tip was used to create a straight scratch wound. The same wounded areas were imaged at 0 and 24 h, respectively. The migration of ECs was also determined by the Transwell migration assay. In brief, the HUVEC pre-treated with different TAMs-CM were suspended in the serum-free medium and plated into the upper chamber of Transwell. Fresh medium containing 15% FBS was added into the wells. After 24 h, the cells that migrated into the basolateral side of the upper chambers were fixed with 4% (w/v) PFA and stained with crystal violet. The migrated cells were counted and photographed using a microscope.

### Tube formation assay

Tube formation assay was performed as previously described with minor modifications 33. In brief, the Matrigel was thawed overnight at 4 °C in advance. On the next day, the Matrigel-coated plates were placed in the incubator and incubated for 45 min. The prepared HUVECs cell suspension was added to the plates and incubated in the incubator. The images of tube formation were acquired with a microscope at 2-6 h following the incubation.

### Statistical analysis

Data were presented as mean ± SD from more than three independent experiments. Student's *t -* test was used for comparison between the two groups, One-way ANOVA was used for comparison between multiple groups, and statistical significance was determined at *p* < 0.05 by using GraphPad Prism 9.0.0) software.

## Results

### Elevated expression of GLUT1 is closely associated with exacerbated tumor progression

**Figure 1:**
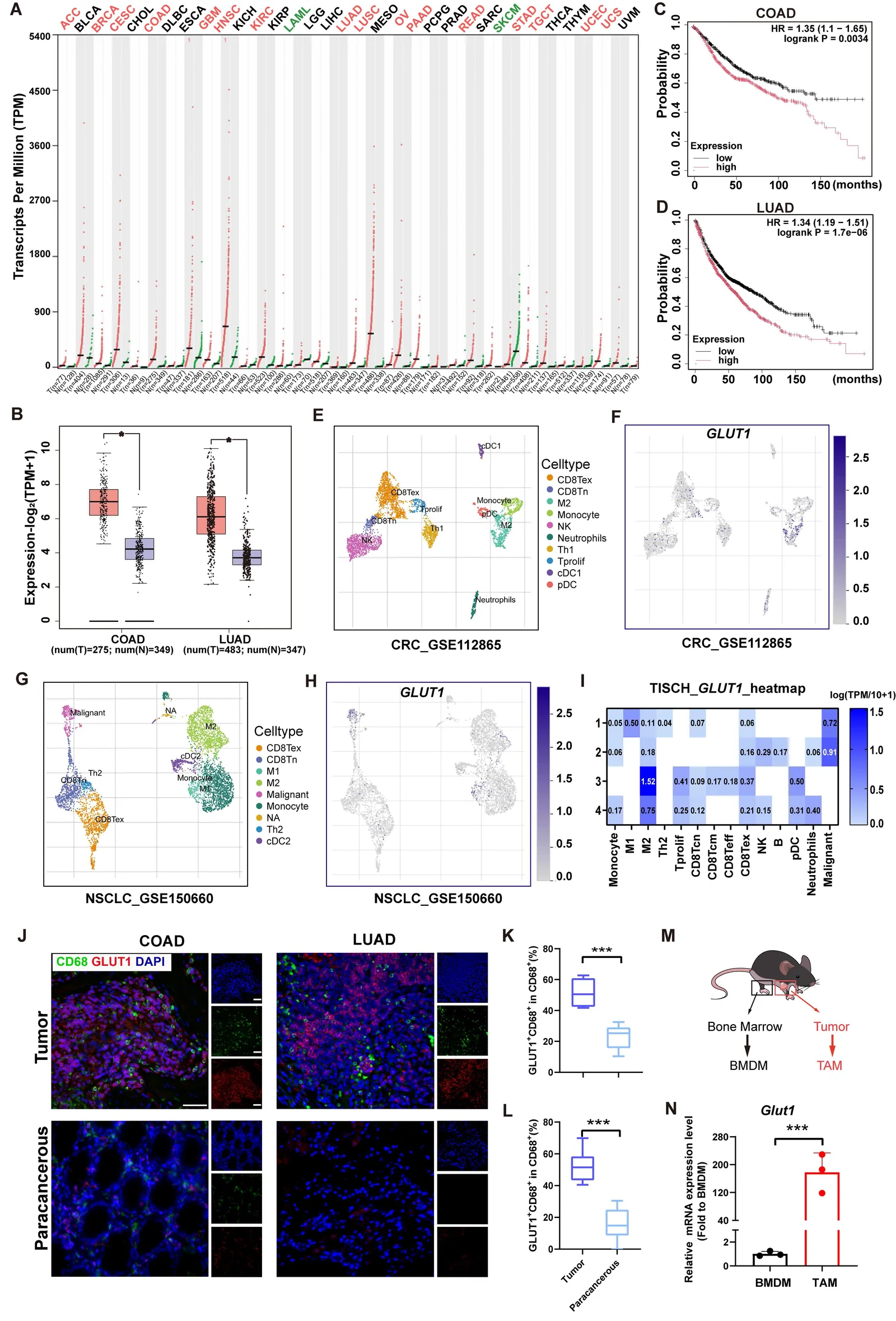
** Elevated expression of GLUT1 is closely associated with exacerbated tumor progression.** (A) GEPIA2 database analysis for GLUT1 expression profile across numerous types tumor samples and paired normal tissues. (B) GEPIA2 database analysis for the tissue-specific expression of GLUT1 in colon adenocarcinoma (COAD) and lung adenocarcinoma (LUAD) (Match TCGA normal and GTEx data). (C and D) Kaplan-Meier Plotter analysis for the overall survival (OS) of COAD and LUAD patients with high expression or low expression of GLUT1. (E) Cell clustering analysis of scRNA-seq data in the CRC_GSE112865_mouse_aPD1 dataset. (F) The expression of *Glut1* gene in different types of cells in the CRC_GSE112865_mouse_aPD1 dataset. (G) Cell clustering analysis of scRNA-seq data in the NSCLC_GSE150660 dataset. (H) The expression of *GLUT1* gene in different types of cells. (I) Heatmap of *GLUT1* gene expression in different types of cells in four data sets, 1 is CRC_GSE112865, 2 is CRC_GSE122969, 3 is NSCLC_GSE150660 and 4 is NSCLC_GSE127465. (J) Human tumor samples were collected from patients with COAD (n =8) or LUAD (n =8). The immunostaining of CD68, GLUT1, and DAPI was performed. Representative IF images for CD68, GLUT1, and DAPI are shown (scale bars: 100 μm). Quantification of percentages of GLUT1^+^CD68^+^ cells in the CD68^+^ cells in the patients with COAD (K) and LUAD (L). (M) Schematic diagram of obtaining mouse BMDMs and TAMs. (N) Quantification for the mRNA expression levels of *Glut1* in the BMDM and TAMs. The data were presented as mean ± SD and analyzed by student's *t* test. * *p*< 0.05, ** *p* < 0.01 and *** *p* < 0.001.

In order to investigate the role of GLUT1 in tumor progression, we initially utilized GEPIA2 34 (http://gepia2.cancer-pku.cn/) to analyze the expression of *GLUT1* in the cancer patients from The Cancer Genome Atlas (TCGA) cohorts and The Genotype-Tissue Expression (GTEx), and it was found that the mRNA expression of *GLUT1* was significantly upregulated in multiple types of tumors as compared to the corresponding para-carcinoma normal tissues, including ACC (adrenocortical carcinoma), BRCA (breast invasive carcinoma), CESC (cervical squamous cell carcinoma and endocervical adenocarcinoma), COAD (colon adenocarcinoma), GBM (glioblastoma multiforme), HNSC (head and neck squamous cell carcinoma), KIRP (kidney renal papillary cell carcinoma), LUAD (lung adenocarcinoma), LUSC (lung squamous cell carcinoma), OV (ovarian serous cystadenocarcinoma), PAAD (pancreatic adenocarcinoma), READ (rectum adenocarcinoma), STAD (stomach adenocarcinoma), TGCT (testicular germ cell tumors), UCEC (uterine corpus endometrial carcinoma) and USC (uterine carcinosarcoma ) (Fig. 1A and 1B). Moreover, Kaplan-Meier Plotter analysis 35, 36 (https://kmplot.com/analysis/) unveiled that high mRNA expression of *GLUT1* was tightly correlated with reduced overall survival (OS) of the COAD and LUAD patients (Fig. 1C and 1D).

To further determine the expression level of GLUT1 in tumors at the single-cell level, we thus performed the single-cell RNA sequencing (scRNA-seq) analysis via using the Tumor Immune Single-Cell Hub (TISCH) datasets 37, 38 (http://tisch.comp-genomics.org/). The analyzed data were derived from the four cancer datasets including CRC_GSE112865_mouse_aPD1 39, CRC_GSE122969_mouse_aPD1aTIM3 40, NSCLC_GSE150660 41 and NSCLC_GSE127465 42. To this end, we examined the expression of GLUT1 in different types of cells based on these datasets, and it was observed that GLUT1 was extensively expressed in the macrophages of colorectal cancer (CRC) and non-small cell lung cancer (NSCLC) (Fig. 1E-1I, Fig. S1A-S1D). To clinically validate these findings, a retrospective analysis of patient-derived tissue samples was conducted accordingly. Consistently, the expression of GLUT1 in macrophages was significantly higher in the tumor tissues of COAD patients compared to that in the paracancerous tissues (Fig. 1J and 1K). Furthermore, the density of CD68⁺ macrophages with high expression of GLUT1 was remarkably elevated within tumor regions relatively to paracancerous tissues. A similar pattern was visualized in the tumor samples of LUAD patients, which displayed prominently greater abundance of GLUT1-high CD68⁺ macrophages in the tumor regions as compared to the normal adjacent tissues (Fig. 1J and 1L). The above findings were consistent with the study by Shi *et al*. 18, reporting that GLUT1 was exclusively and highly expressed in M2-like TAMs in B16 melanoma and LLC tumors, and that these M2-like TAMs exhibited high glucose uptake capacity.

Additionally, in order to further validate the expression of GLUT1 in the macrophages, we thus established the subcutaneous MC38 tumor-bearing mouse model, and subsequently isolated the macrophages from the mouse bone marrow and tumor tissues, respectively (Fig. 1M). Intriguingly, it was revealed that the mRNA expression of *Glut1* was significantly upregulated in the TAMs compared with that in the BMDMs (Fig. 1N). Collectively, these results suggest that the elevated expression of GLUT1 in tumor tissues, particularly within TAMs, is closely associated with exacerbated tumor progression.

### Macrophage-specific depletion of Glut1 gives rise to resistance to tumor growth

**Figure 2:**
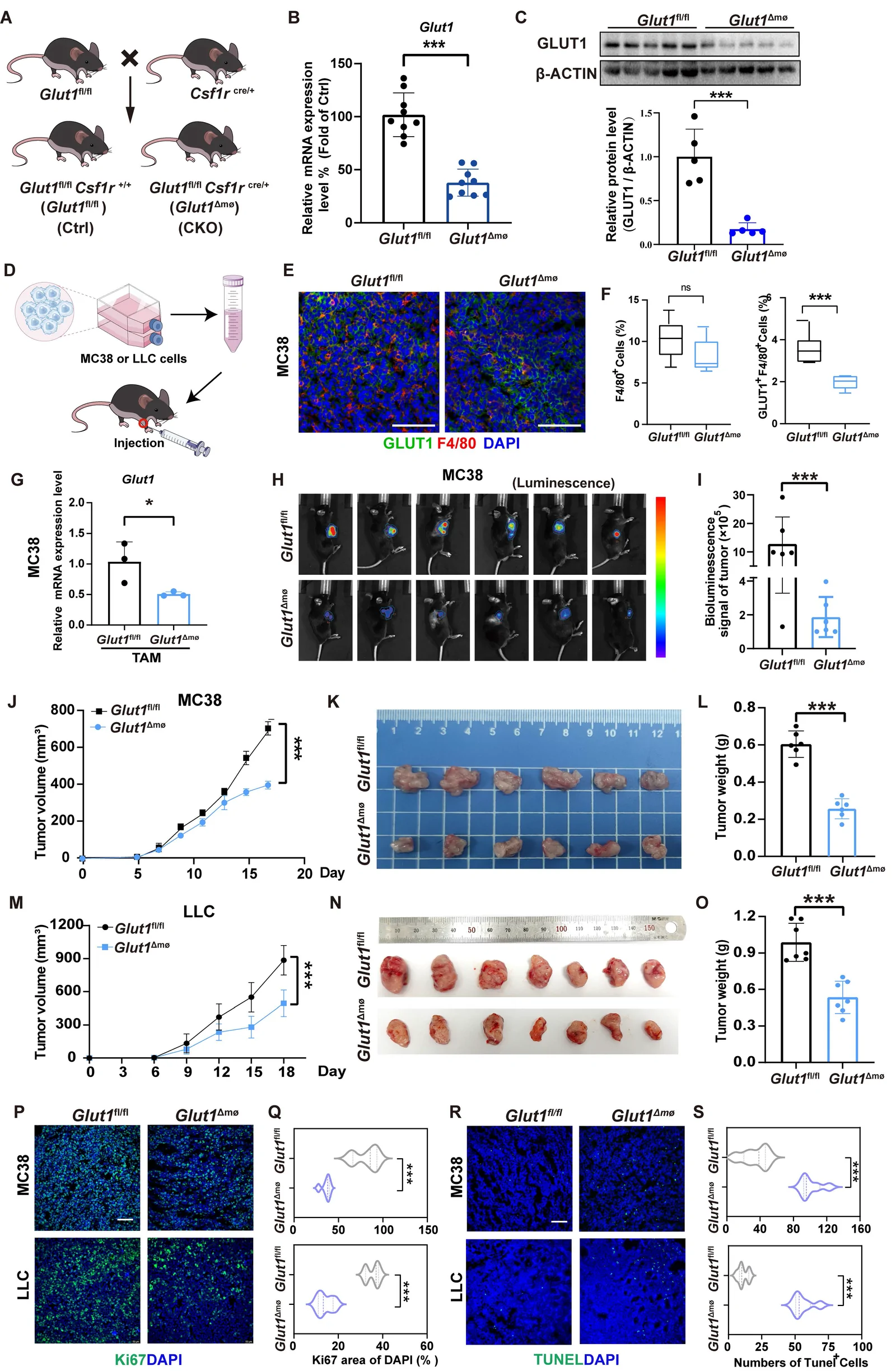
** Macrophage-specific depletion of Glut1 gives rise to resistance to tumor growth.** (A) Schematic diagram of generation of macrophage-specific *Glut1* knockout mice. (B) Quantification for the mRNA expression levels of *Glut1* in the BMDMs derived from* Glut1*^Δmø^ and* Glut1*^fl/fl^ mice. (C) Protein expression levels of GLUT1 in the BMDMs derived from *Glut1*^fl/fl^ and* Glut1*^Δmø^ mice (n = 5). (D) Schematic diagram of the mouse subcutaneous tumor model. (E) Representative IF images for co-staining of GLUT1 (green), F4/80 (red) and DAPI (blue) in the MC38 tumor-bearing mice (scale bars: 100 μm). (F) Quantification for the percentage of F4/80^+^ macrophages and GLUT1^+^F4/80^+^ macrophages in the total F4/80^+^ macrophages. (G) The mRNA expression of *Glut1* in the TAMs derived from *Glut1*^fl/fl^ and *Glut1*^Δmø^ mice. (H and I) Bioluminescence images and quantitative analysis of the MC38 tumors in the *Glut1*^fl/fl^ and *Glut1*^Δmø^ mice. (J) Tumor growth curves showing the mean tumor volume at the indicated timepoints following the implantation of MC38 cells into mice (n = 7). (K) Image of the tumors harvested at day 17 after MC38 cell inoculation. (L) Quantification of weights of tumors harvested at day 17 after MC38 cell inoculation. (M) Tumor growth curves showing the mean tumor volume at the indicated time points following the implantation of LLC cells into mice (n = 7). (N) Image of the tumors harvested at day 18 after LLC cell inoculation. (O) Quantification of weights of tumors harvested at day 18 after LLC cell inoculation. (P) Representative IF staining of Ki67 in the tumor tissues (scale bars: 100 μm). (Q) Quantifications for the percentages of Ki67^+^ in the DAPI^+^ cells. (R) Representative IF staining of TUNEL staining in the tumor tissues (scale bars: 100 μm). (S) Quantifications for the percentages of TUNEL^+^ in the DAPI^+^ cells. The data were presented as the mean ± SD and analyzed by student's *t* test. * *p*< 0.05, ** *p* < 0.01 and *** *p* < 0.001.

To next investigate the biological functions of GLUT1 expressed in the TAMs in the process of tumor development, we generated the macrophage-specific GLUT1 KO mice by using the Cre-loxP system 43. More specifically, *Glut1*^fl/fl^*Csf1r*^cre/+^ mice were used as the conditional KO group (CKO; *Glut1*^Δmø^ mice), while *Glut1*^fl/fl^ *Csf1r*^+/+^ mice were utilized as the control group (*Glut1*^fl/fl^ mice) (Fig. 2A). The KO efficiency of GLUT1 in macrophages was validated by performing the RT-qPCR analysis for BMDMs from the *Glut1*^Δmø^ and *Glut1*^fl/fl^ mice. Notably, the mRNA expression of GLUT1 in the BMDMs was dramatically lower in the *Glut1*^Δmø^ mice compared to *Glut1*^fl/fl^ mice (Fig. 2B). In parallel, the protein level of GLUT1 in the BMDMs derived from the *Glut1*^Δmø^ mice was also substantially reduced compared to that from the *Glut1*^fl/fl^ mice (Fig. 2C).

To further explore the role of macrophagic GLUT1 in influencing the progression of tumors, we established two different syngeneic tumor models in which MC38 or LLC cells were inoculated subcutaneously into the *Glut1*^fl/fl^ and *Glut1*^Δmø^ mice (Fig. 2D). Taking advantage of the MC38 CRC model, IF staining was performed on the harvested tumor tissues. As expected, GLUT1 protein expression in F4/80^+^ macrophages within tumor tissues was significantly lower in the *Glut1*^Δmø^ mice than that in the *Glut1*^fl/fl^ mice (Fig. 2E and 2F), whereas there was no significant difference in light of the percentage of infiltrated total F4/80^+^ macrophages between these two groups (Fig. 2F). Furthermore, the TAMs (CD45^+^CD11b^+^F4/80^+^) were sorted out using flow cytometry from the tumor-bearing mice. In line with the IF staining data, it was illustrated that the mRNA expression level of *Glut1* was robustly lower in the TAMs derived from the *Glut1*^Δmø^ mice than that in the TAMs derived from the *Glut1*^fl/fl^ mice (Fig. 2G).

Of interest, the growth of MC38 tumors was strikingly hampered in the *Glut1*^Δmø^ mice compared to that in the *Glut1*^fl/fl^ mice, as evidenced by the *in vivo* bioluminescence imaging (Fig. 2H and 2I). Moreover, we consistently observed that both tumor volume and tumor weight were significantly lower in the macrophage-specific *Glut1*-KO mice than those in the control mice (Fig. 2J-2L). Consistently, in the LLC tumor model, *Glut1*^Δmø^ mice also exhibited profoundly slower tumor growth and smaller tumor volumes compared to the *Glut1*^fl/fl^ mice (Fig. 2M-2O). We subsequently determined the tumor biological behaviors accordingly. Intriguingly, the expression of Ki67 was prominently decreased (Fig. 2P and 2Q) and TUNEL positivity was strikingly increased (Fig. 2R and 2S) in the tumor tissues of *Glut1*^Δmø^ mice compared to that of the *Glut1*^fl/fl^ mice in both MC38 and LLC tumor-bearing mice, which indicated that the proliferation of tumor cells was suppressed and the apoptosis of tumor cells was enhanced upon the deletion of GLUT1 in macrophages. Taken together, our data suggest that depletion of GLUT1 in macrophages is prone to retard the progression of tumors in mice.

### Macrophage-specific deletion of Glut1 strengthens the tumor immune microenvironment

**Figure 3:**
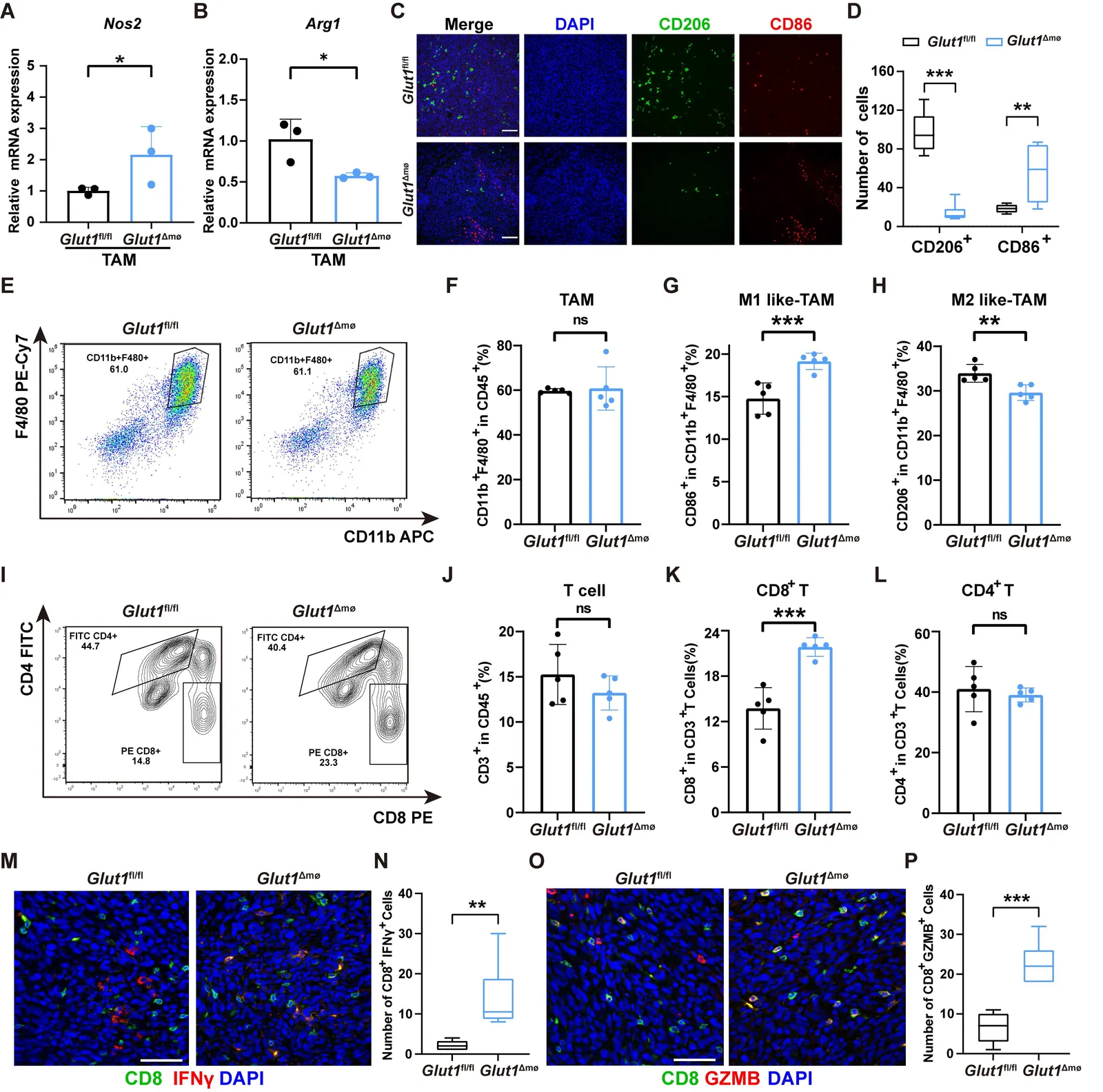
** Macrophage-specific deletion of Glut1 strengthens the tumor immune microenvironment.** (A) The mRNA expression of *Nos2(Inos)* in the TAMs derived from *Glut1*^fl/fl^ and* Glut1*^Δmø^ mice. (B) The mRNA expression of *Arg1* in the TAMs derived from *Glut1*^fl/fl^ and* Glut1*^Δmø^ mice. (C) Representative IF images of co-staining for CD206 (green), CD86 (red) and DAPI (blue) in the MC38 tumor-bearing mice (scale bars: 100 μm). (D) Quantification for the number of CD206^+^ or CD86^+^ cells. (E) Flow cytometry analysis of total macrophages (CD45^+^CD11b^+^F4/80^+^). Quantitative graphs of (F) total macrophages (CD45^+^CD11b^+^F4/80^+^), (G) M1-like macrophages (CD45^+^CD11b^+^F4/80^+^CD86^+^), and (H) M2-like macrophages (CD45^+^CD11b^+^F4/80^+^CD206^+^) in the tumor tissues (n = 5). (I) Flow cytometry analysis of total T cells (CD45^+^CD3^+^). Quantitative graphs of total (J) T cells (CD45^+^CD3^+^), (K) CD8^+^ T cells (CD45^+^CD3^+^CD8^+^), and (L) CD4^+^ T cells (CD45^+^CD3^+^CD4^+^) in the tumor tissues (N = 5); (M) Representative IF images for co-staining of CD8^+^ (green) and IFNγ (red) in tumors (scale bars: 50 μm). (N) Quantification for the number of CD8^+^IFNγ^+^ T cells. (O) Representative IF images for co-staining of CD8^+^ (green) and GZMB (red) in tumors (scale bars: 50 μm). (P) Quantification for the number of CD8^+^GZMB^+^ T cells. The data were presented as the mean ± SD and analyzed by student's *t* test. * *p*< 0.05, ** *p* < 0.01 and *** *p* < 0.001.

Aiming to elucidate how macrophage GLUT1 shapes the TIME, we next assessed its effects on macrophage polarization and function. Indeed, different subtypes of macrophages tend to display conspicuous functional differences, M1-like macrophages are deemed to exert anti-tumor responses by virtue of various mechanisms, whereas M2-like macrophages are inclined to accelerate tumor growth and metastasis via eliciting angiogenesis and immunosuppression 44. To this end, we next dissected the functions of *Glut1* expression on polarized subtypes of macrophages. As shown in Figure 3A and 3B, we found that the mRNA expression of *Inos* (considered as a representative M1 marker) was significantly higher but the mRNA expression of *Arg1* (regarded as a classic M2 marker) was dramatically lower in the *Glut1*^Δmø^ mice than that in the *Glut1*^fl/fl^ mice, which implicated that M2 polarization was rigorously restrained in the *Glut1*-deficient macrophages. Moreover, IF staining results revealed a salient increase in the number of CD86⁺ macrophages but an obvious decrease in the number of CD206⁺ macrophages in the *Glut1*^Δmø^ mice relative to *Glut1*^fl/fl^ mice (Fig. 3C and 3D). These data were further substantiated by the flow cytometry analysis despite no significant difference in the total number of infiltrated macrophages between the two groups (Fig. 3E-3H). Similar findings were obtained in the LLC lung cancer model, in which the data showed that macrophage-specific GLUT1 deficiency failed to alter the infiltration of total TAMs but remarkably shifted their polarization profile (Fig. S2A-S2F), as evidenced by an increase in the M1-like macrophages but a decrease in M2-like macrophages in the *Glut1*^Δmø^ mice compared to *Glut1*^fl/fl^ mice (Fig. S2G-S2I).

It has been widely held that T cells serve as the core immune effector cells in the TME. Tumor-infiltrating T cells are capable of directly killing tumor cells or secreting numerous cytokines to modulate the TIME through recognizing tumor-specific antigens 45. Among them, CD8^+^ cytotoxic T cells are thought to directly give rise to tumor cell apoptosis 46. Activated CD8^+^ T cells are prone to boost their own cytotoxicity, efficiently kill tumor cells as well as enhance the body's immune response ability via releasing increased amount of interferon-γ (IFN-γ) and granzyme B (GZMB) 47-49. As such, we further examined whether GLUT1 expression in macrophage could have any impacts on T cells in the TME. Of note, in the MC38 tumor-bearing mice, the infiltration of total T cells (CD45^+^CD3^+^) or CD4^+^ T cells remained unchanged between the *Glut1*^Δmø^ and *Glut1*^fl/fl^ mice, but the number of infiltrated CD8^+^T cells was prominently elevated in the *Glut1*^Δmø^ mice compared to that in the *Glut1*^fl/fl^ mice (Fig. 3I-3L). Homoplastically, the absence of GLUT1 in the macrophages resulted in an elevated infiltration of CD8^+^ T cells into the tumors in the LLC tumor-bearing mice (Fig. S2J-S2L). More interestingly, the numbers of both IFN-γ^+^CD8^+^ T cells and GZMB^+^CD8^+^ T cells were strikingly enhanced in the *Glut1*^Δmø^ mice compared to that in the *Glut1^fl/fl^* mice (Fig. 3M-3P; Fig. S2J-S2N), which implied that the mice with deficiency of macrophagic GLUT1 demonstrated strengthened anti-tumor immune responses.

### Macrophage-specific deletion of Glut1 limits angiogenesis and normalizes blood vessels in tumors

**Figure 4:**
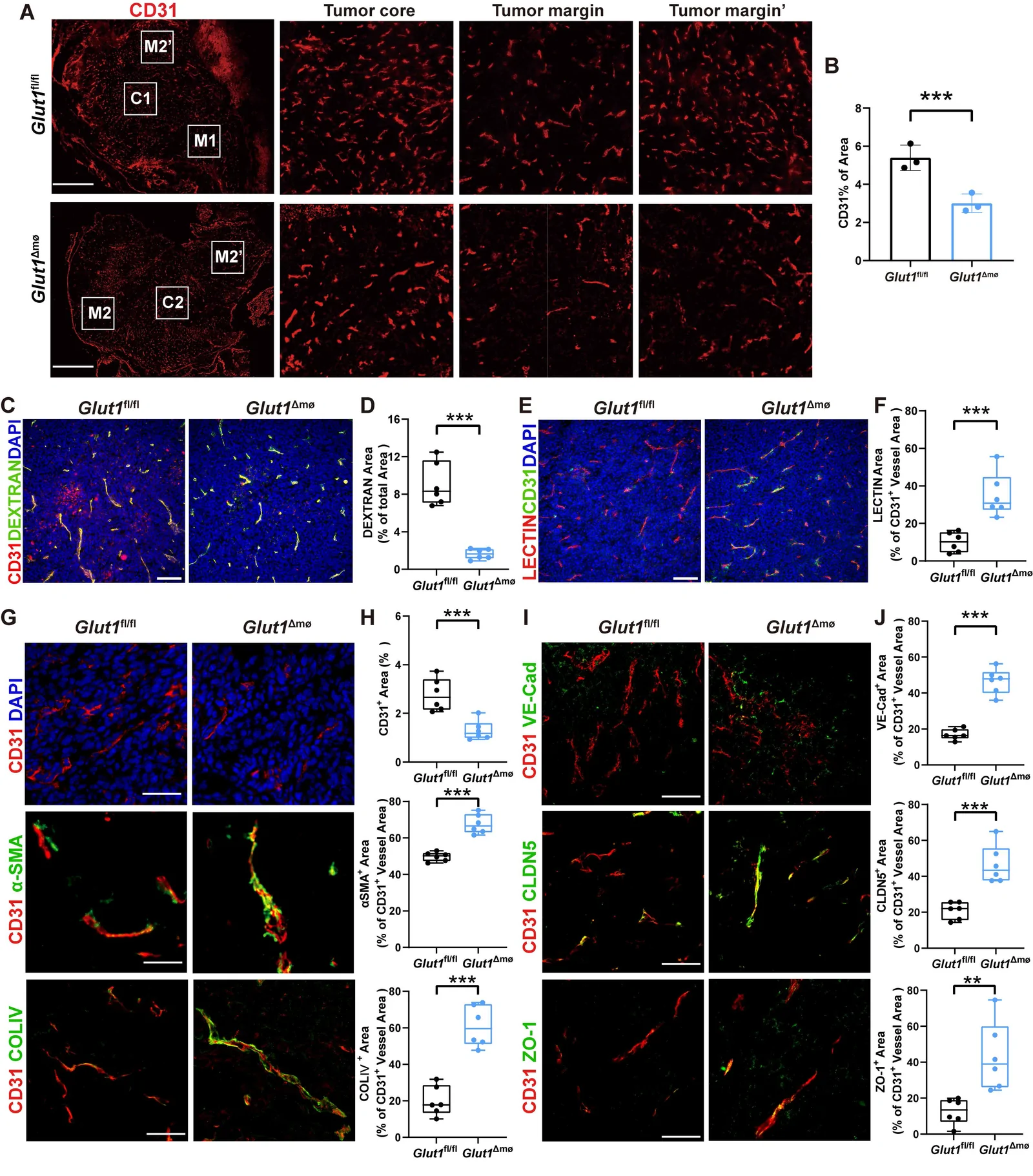
** Macrophage-specific deletion of Glut1 limits angiogenesis and normalizes blood vessels in tumors.** (A) Representative IF images for CD31 staining of the whole tumors harvested from the *Glut1*^fl/fl^ and* Glut1*^Δmø^ mice (scale bars: 1 mm). (B) Quantification for the percentage of CD31^+^ area in the tumors harvested from the *Glut1*^fl/fl^ and* Glut1*^Δmø^ mice. (C) Representative IF images of leaked dextran (green) in the tumor blood vessels (stained with CD31), scale bars: 100 μm. (D) Quantification for the percentage of dextran^+^ area around the vessel area (n = 6). (E) Representative IF images for the perfused lectin (red) in the tumor blood vessels (stained with CD31), scale bars: 100 μm. (F) Quantification for the percentage of lectin^+^ area in the vessel area (n = 6). (G) Representative IF images for co-staining of CD31 (red) with DAPI (blue), α-SMA (green) or COL IV (green) in the tumor tissues (scale bars: 50 μm). (H) Quantification for the number of blood vessels, as well as the percentage of CD31^+^ area, CD31^+^α-SMA^+^ area and CD31^+^COL IV^+^ area (n = 6). (I) Representative IF images for co-staining of CD31 (red) with VE-Cad (blue), CLDN5 (green) or ZO-1 (green) in the tumor tissues (scale bars: 50 μm). (J) Quantification for the percentage of CD31^+^VE-Cad^+^ area, CD31^+^CLDN5^+^ area and CD31^+^ZO-1^+^ area (n = 6). The data were presented as the mean ± SD and analyzed by student's *t* test. * *p*< 0.05, ** *p* < 0.01 and *** *p* < 0.001.

To gain insight into the altered biological functions of macrophage-specific depletion of *Glut1* during tumor development, we thus performed transcriptome sequencing (RNA-seq) on the TAMs. On one hand, TAMs were isolated by virtue of fluorescence activated cell sorting (FACS) from the MC38 tumor-bearing *Glut1*^fl/fl^ and *Glut1*^Δmø^ mice (Fig. S3A). On the other hand, an *in vitro* model of GLUT1 knockdown in TAMs was constructed using tumor cell supernatants in combination with lentivirus-mediated shRNA interference (Fig. S3B). Further, the knockdown efficiency of GLUT1 was validated by western blot analysis (Fig. S3C and S3D). RT-qPCR analysis revealed that TCM treatment alone led to decreased expression of M1 markers (*Nos2, Cd86*) and increased expression of M2 markers (*Cd206, Arg1*). Importantly, upon GLUT1 knockdown, macrophages exhibited a reversal of this trend, with significant upregulation of M1 markers and striking downregulation of M2 markers (Fig. S3E-S3H), which was consistent with the macrophage polarization observed previously in tumors (Figure 3). Transcriptomic analysis was conducted on the TAMs in both models. Intriguingly, Gene Ontology (GO) enrichment analysis of differentially expressed genes (DEG) elucidated that GLUT1 expression in macrophages was prone to influence the tumor angiogenesis-related pathways (Fig. S3I and S3J). IF analysis of the tumor tissues collected from the MC38 tumor-bearing mice revealed a prominent reduction in the density of CD31⁺ blood vessels in the *Glut1*^Δmø^ mice compared with *Glut1*^fl/fl^ mice (Fig. 4A and 4B).

It has been increasingly recognized that the abnormal vascular structure in tumors is predominantly featured with insufficient coverage of smooth muscle cells (SMCs) and pericytes, aberrant basement membrane support, as well as disrupted EC junctions. These defects in tumor vascular structure are frequently accompanied by functional impairments in the tumor blood vessels, including reduced vascular perfusion and aggravated vascular leakage 31, 32, 50. Subsequently, we interrogated whether the structural alterations in tumor blood vessels induced by macrophage-specific deletion of *Glut1* were able to improve tumor vascular function. As anticipated, *Glut1*^Δmø^ mice exhibited a profound reduction in the leakage of TRITC-dextran from the blood vessels compared to the *Glut1*^fl/fl^ mice (Fig. 4C and 4D). More intriguingly, it was demonstrated that there were limited blood vessels with Lectin (DyLight488) positive staining in the *Glut1*^fl/fl^ mice, whereas the Lectin positive area in the blood vessels of the *Glut1*^Δmø^ mice was fundamentally escalated, implicating that macrophage-specific deletion of *Glut1* enhanced vascular perfusion in the MC38 tumor-bearing mice (Fig. 4E and 4F). Next, we conducted a detailed analysis for the blood vessels in the MC38 tumors, and it was observed that there was a significant reduction in terms of the density of blood vessels (as indicated by CD31 staining) in the *Glut1*^Δmø^ mice compared to the *Glut1*^fl/fl^ mice, implying that macrophage-specific depletion of GLUT1 restricted tumor angiogenesis (Fig. 4G and 4H). To further determine the associations between TECs and mural cells in tumors, α-SMA and NG2 were utilized to label the SMCs and pericytes, respectively. Surprisingly, co-staining analysis for CD31 and α-SMA highlighted that there was a remarkable elevation in the coverage of SMCs along the blood vessels in the *Glut1*^Δmø^ mice than that in the *Glut1*^fl/fl^ mice (Fig. 4G and 4H). In addition, collagen IV (Col IV)—a critical component of the basement membrane around blood vessels—displayed strikingly improved coverage following macrophage-specific deletion of *Glut1*, indicative of enhanced basement membrane support and structural integrity (Fig. 4G and 4H). Likewise, in comparison to the *Glut1*^fl/fl^ mice, *Glut1*^Δmø^ mice exhibited the increased expression levels of an array of crucial endothelial junctional molecules in the blood vessels including VE-cadherin, ZO-1, and Claudin-5, thereby robustly reinforcing vascular junctional integrity (Fig. 4I and 4J). In concomitant with these findings, LLC tumors also displayed analogous characteristics of vascular normalization upon macrophage-specific deletion of GLUT1. These features included diminished vascular density (Fig. S4A and S4B), enhanced pericyte coverage improved basement membrane support (Fig. S4C and S4D), as well as intensified endothelial junctions, as evidenced by the boosted expression levels of VE-cadherin (VE-Cad), ZO-1, and Claudin-5 (CLDN5) (Fig. S4E and S4F).

**Figure 5:**
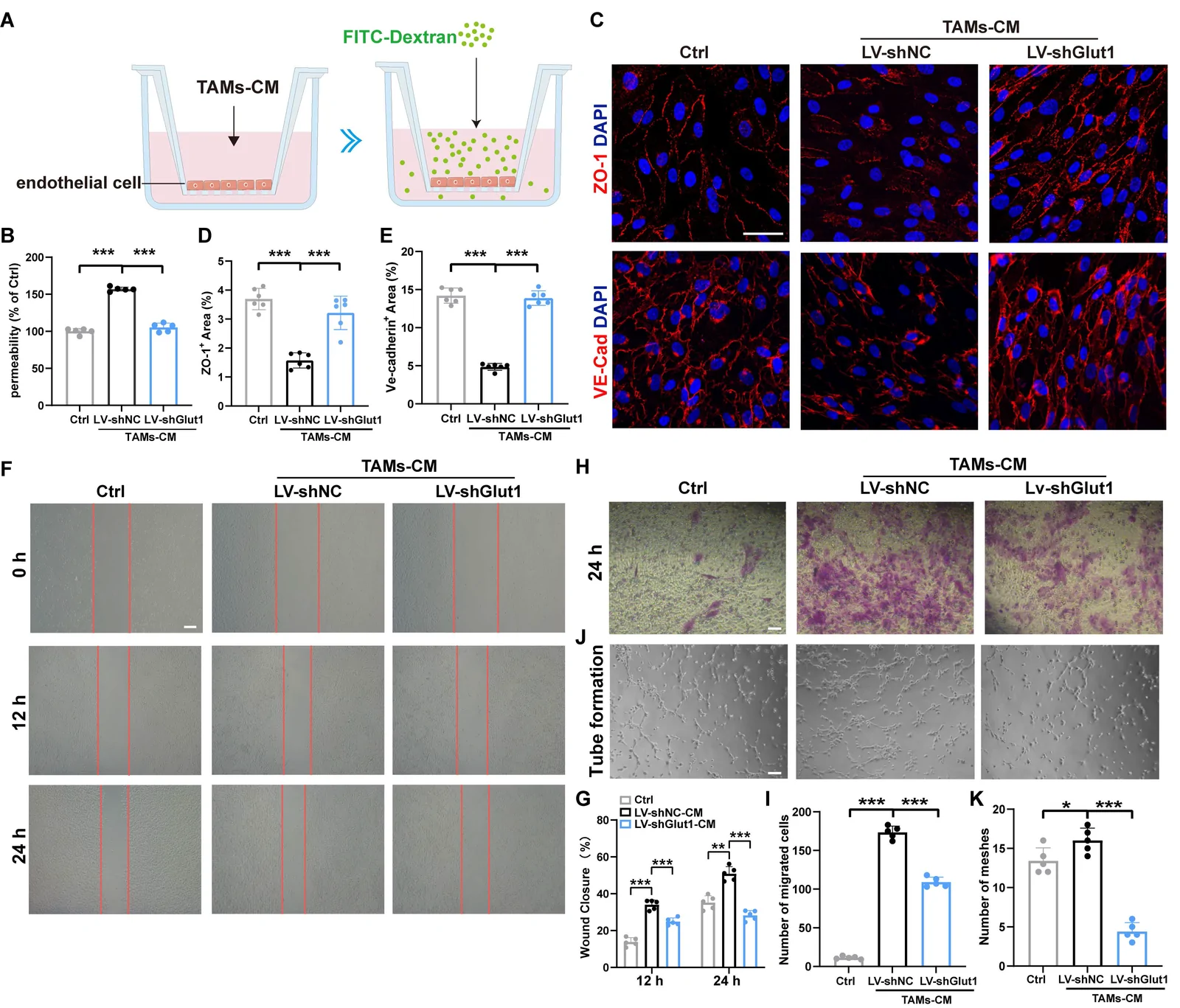
** Knockdown of Glut1 in macrophages inhibits angiogenesis and improves the integrity of ECs.** (A) Schematic diagram of FITC-dextran leakage across endothelial monolayer in the Transwell system. (B) Quantification of the leaked FITC-dextran across the endothelial monolayer in different groups. (C) Representative IF images for the co-staining of ZO-1 (green)/DAPI (blue) or VE-Cad (red)/DAPI (blue) in the HUVEC (scale bars: 50 μm). Quantification of the percentage of VE-Cad^+^ area (D) or ZO-1+ area (E) in the visual field (n = 6). (F) Evaluation of the migration of HUVEC by wound healing assay (scale bars: 200 μm). (G) Quantitative analysis of wound healing assay. (H) Evaluation of HUVECs migration by transwell assay (scale bars: 50 μm). (I) Quantitative analysis of the Transwell migration assay. (J) Evaluation of tube formation ability of HUVEC stimulated by the supernatants from TAMs in different groups (scale bars: 100 μm). (K) Quantitative analysis of tube formation of HUVECs in different groups. The data were presented as the mean ± SD and analyzed using one-way ANOVA or two-way ANOVA. * *p*< 0.05, ** *p* < 0.01 and *** *p* < 0.001.

Furthermore, we used RAW264.7 cells (mouse mononuclear macrophage leukemia cells) and HUVECs to establish an *in vitro* model and subsequently investigated the impacts of macrophagic GLUT1 expression on the biological behaviors of ECs. To assess the endothelial barrier function, we utilized a Transwell system where an endothelial monolayer was constructed in the upper chamber, and the dextran dye was added to the apical side of the monolayer to measure its leakage into the lower chamber. To our surprise, it was elucidated that TAMs-conditioned media (TAMs-CM) tended to profoundly increase the leakage of dextran and thus elevate endothelial permeability. Nevertheless, the CM derived from the TAMs with silence of GLUT1 potently alleviated endothelial leakage and improved endothelial integrity (Fig. 5A and 5B). Moreover, control TAMs-CM contributed to conspicuous downregulation of the tight junctions (TJs)-associated proteins including ZO-1 and VE-cadherin in the ECs. In contrast, the CM derived from the TAMs with knockdown of GLUT1 was inclined to restore the expression levels of these proteins and improve TJ integrity of ECs (Fig. 5C-5E). Further, we also examined the effect of TAMs-CM on the migration of ECs by virtue of wound healing and Transwell migration assays. Notably, ECs treated with control TAMs-CM displayed dramatically escalated migratory capability compared to the blank control group, which was markedly reversed by the CM derived from TAMs with knockdown (Fig. 5F-5I). Additionally, tube formation assay uncovered that HUVECs treated with GLUT1-knockdown TAMs-CM exhibited a strikingly diminished capacity to produce capillary-like structures as compared to those under exposure to control TAMs-CM (Fig. 5J-5K). In summary, all of these findings implicate that silence of GLUT1 in TAMs restricts the migration and tube formation of ECs, attenuates vascular leakage, and strengthens intercellular junctions, thereby improving endothelial barrier function and fortifying structural stability.

Aberrant tumor vasculature creates a hostile microenvironment characterized by hypoxia, acidosis, poor perfusion, and immunosuppressive cytokine release, which collectively impede the infiltration of CD8⁺ T cells and other cytotoxic effectors 51. Conversely, vascular normalization reverses these deficits, enhancing immune cell infiltration, bolstering immunotherapeutic responses, and maximizing drug delivery efficiency 52. Therefore, the vascular normalization phenotype observed following GLUT1 depletion in TAMs may partially contribute to the enhanced anti-tumor immune response identified in our study.

### Loss of Glut1 in macrophages limits the secretion of multiple inflammatory mediators through regulating the GSK3β/β-catenin/CD36 signaling axis

**Figure 6:**
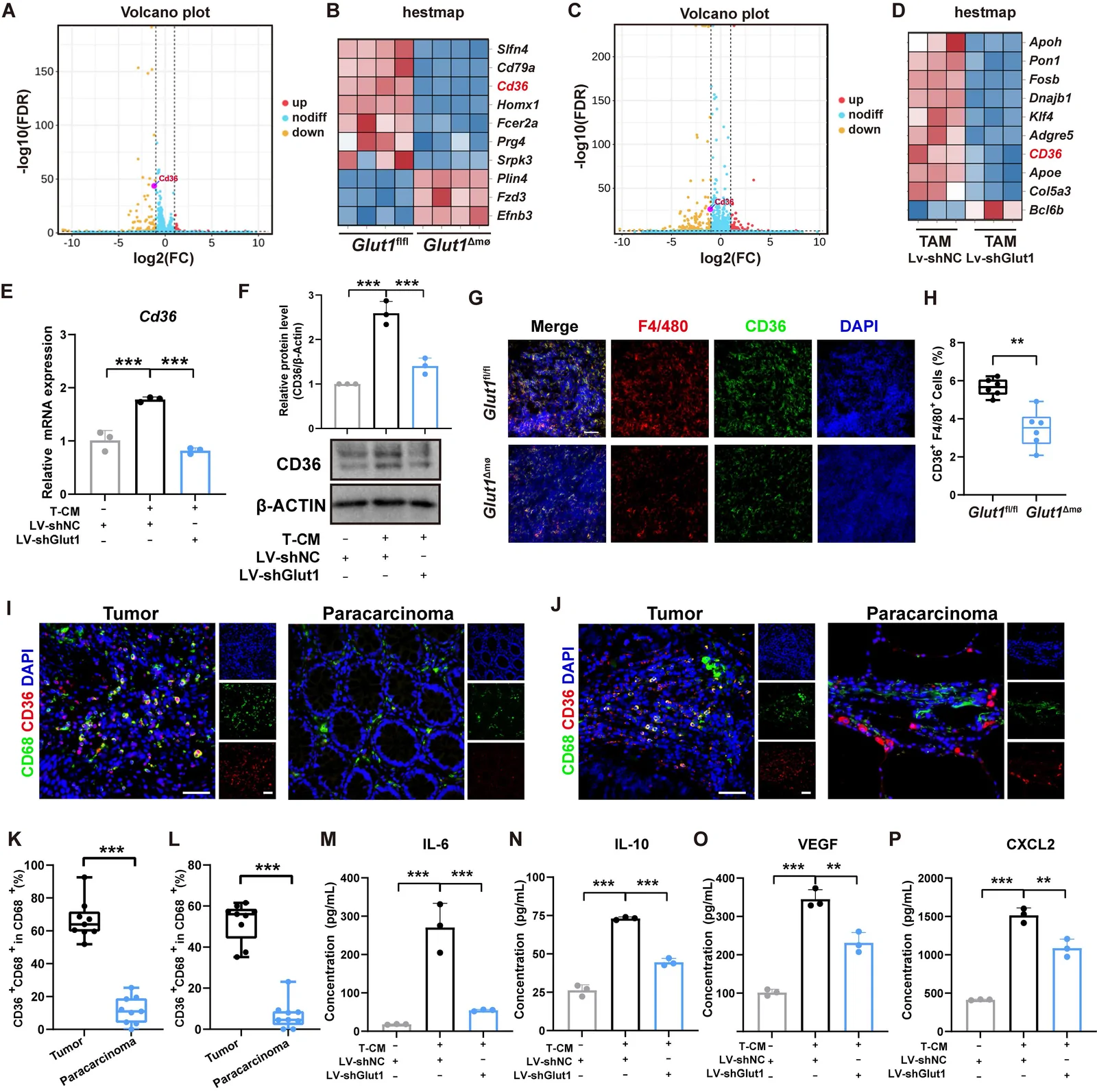
** GLUT1 in macrophages is able to regulate the expression of CD36.** (A and B) RNA-seq analysis of TAMs in MC38 tumor-bearing mice. Volcano map (A) for differential analysis and heatmap (B) of the top 10 differentially expressed genes between *Glut1*^fl/fl^ and* Glut1*^Δmø^ mice. (C and D) RNA-seq of RAW264.7 cells following the intervention of tumor cell supernatants. Volcano plot (C) for differential analysis and heatmap (D) of the top 10 differentially expressed genes between Lv-shNC and Lv-shGlut1 TAMs. (E) The mRNA expression levels of *Cd36* in the RAW264.7 cells after the intervention of tumor cell supernatants. (F) Western blot analysis of protein expression of CD36 in the RAW264.7 cells upon different treatments. (G) Representative IF staining images for co-staining of CD36 (green), F4/80 (red) and DAPI (blue) in the MC38 tumor-bearing mice (scale bars: 100 μm). (H) Quantitative analysis for the percentage of CD36^+^F4/80^+^ macrophages in the total F4/80^+^ macrophages (n = 5). (I and J) Human tumor samples were collected from patients with COAD (n =8) or LUAD (n =8). The immunostaining of CD68, CD36, and DAPI was performed. Representative IF images for CD68, CD36, and DAPI in the COAD (I) and LUAD (J) samples are shown (scale bars: 100 μm). (K and L) Quantification of percentages of CD36^+^CD68^+^ cells in the CD68^+^ cells in the patients with COAD (K) and LUAD (L). (M-P) The levels of IL-6 (M), IL-10 (N), VEGF (O) and CXCL2 (P) in the supernatants of RAW264.7 macrophages were measured by ELISA. The data were presented as the mean ± SD and analyzed by student's *t -* test and one-way ANOVA. * *p*< 0.05, ** *p* < 0.01 and *** *p* < 0.001.

In order to gain insight into the mechanisms underlying loss of GLUT1 in macrophage provoked tumor vascular normalization, we further comprehensively dissected the RNA-seq data of TAMs described above. More specifically, we conducted integrated transcriptomic analyses on primary TAMs isolated from mouse tumor tissues (Fig. 6A and 6B) and *in vitro* polarized RAW264.7 macrophages (Fig. 6C and 6D). Strikingly, CD36 was identified as a core overlapping DEG, which consistently ranked among the top ten most significantly changed DEGs in both screening systems (Fig. 6A-6D). Importantly, CD36 expression was markedly downregulated in TAMs from both* in vitro* and *in vivo* systems upon macrophage-specific GLUT1 knockout (Fig. 6A-6D). In fact, CD36 as a scavenger receptor highly expressed on macrophages is deemed to play a pivotal role in macrophage-induced inflammatory responses, immune regulation, and lipid metabolism 53-56.

Of interest, incubation of RAW264.7 cells with TCM led to augmented mRNA expression levels of *Cd36* (Fig. 6E). On the contrary, infection with LV-shGlut1 resulted in a significant reduction in light of the mRNA expression levels of *Cd36* (Fig. 6E). In congruent with these findings, the protein expression levels of CD36 displayed similar alterations (Fig. 6F). Homoplastically, the proportion of CD36⁺F4/80⁺ macrophages were remarkably diminished in tumors from the *Glut1*^Δmø^ mice compared to that from the *Glut1*^fl/fl^ mice in the MC38 tumor-bearing mice (Fig. 6G and 6H). More importantly, retrospective analysis for clinical samples elucidated that there was a prominent increase in the percentage of CD36⁺CD68⁺ macrophages within the primary tumor tissues of patients (Fig. 6I-6L). Overall, these *in vitro* and *in vivo* findings highlight that the expression of CD36 in macrophages can be directly regulated by GLUT1.

It has been highly appreciated that complicated interactions between TAMs and TECs play a critical role within the TME. On one hand, vascular ECs provide a specific microenvironment that propels the differentiation and M2 polarization of macrophages 57, thereby governing the anti-tumor immune responses mediated by T cells and macrophages. On the other hand, TAMs are able to promote tumor angiogenesis via elevated release of multiple pro-angiogenic factors (such as VEGF, PDGF-B, FGF, and EGF) 58, 59, angiogenesis regulatory cytokines including TGF-β and TNF-α, as well as chemokine ligands including CXCL12 and CXCL8 21, 25, 60. By virtue of the reciprocal interactions, TAMs and TECs mutually influence each other and collectively result in the dynamic remodeling of the TME. Notably, CD36 expressed on the surface of macrophages is prone to exert the impacts on the expression of multiple adhesion molecules in ECs via modulating the secretion of various cytokines, such as IL-6 and IL-10 61. This cascade event tends to alter vascular permeability, and regulate tumor angiogenesis and EC function, thereby influencing the dynamic balance of TME 62. CXCL2 is a member of the chemokine family that regulates the chemotaxis of neutrophils and monocytes, contributing to tumor metastasis, angiogenesis, and tissue repair 63. Moreover, the CXCL2/IL-8/CXCR2 signaling axis is able to modulate EC proliferation, migration, and angiogenic activity 64. As such, we collected the supernatants from macrophages for ELISA analysis, and it was observed that macrophages were able to secrete more cytokines including IL-10 (cytokine), IL-6 (cytokine), CXCL2 (chemokine) and VEGF (growth factor) following the incubation of TCM compared to vehicle control. However, silence of GLUT1 in macrophages profoundly blunted the release of these factors (Fig. 6M-6P). Silencing GLUT1 in macrophages significantly upregulated the levels of M1-related cytokines (IL-12, TNF-α) (Fig. S5A- S5B). To further investigate the functional role of CD36, we performed siRNA-mediated knockdown in TAMs (Fig. S5C). Noteworthily, TCM treatment enhanced the levels of VEGF, IL-10, IL-6, and CXCL2, which could be reversed upon the silence of CD36 (Fig. S5D- S5G). These results indicate that CD36 is required for TCM-induced upregulation of these mediators in TAMs, supporting its role as a downstream effector of GLUT1 signaling. Furthermore, treatment of ECs with the supernatants from macrophages unveiled that TAMs-CM was able to upregulate the mRNA expression levels of *Vcam1*, *Icam1*, and *Cd62e* in ECs, indicative of increased endothelial inflammation and permeability. Conversely, the supernatants from macrophages with knockdown of GLUT1 attenuated the expression levels of these adhesion molecules and prevented inflammatory activation (Fig. S5H-S5J).

**Figure 7:**
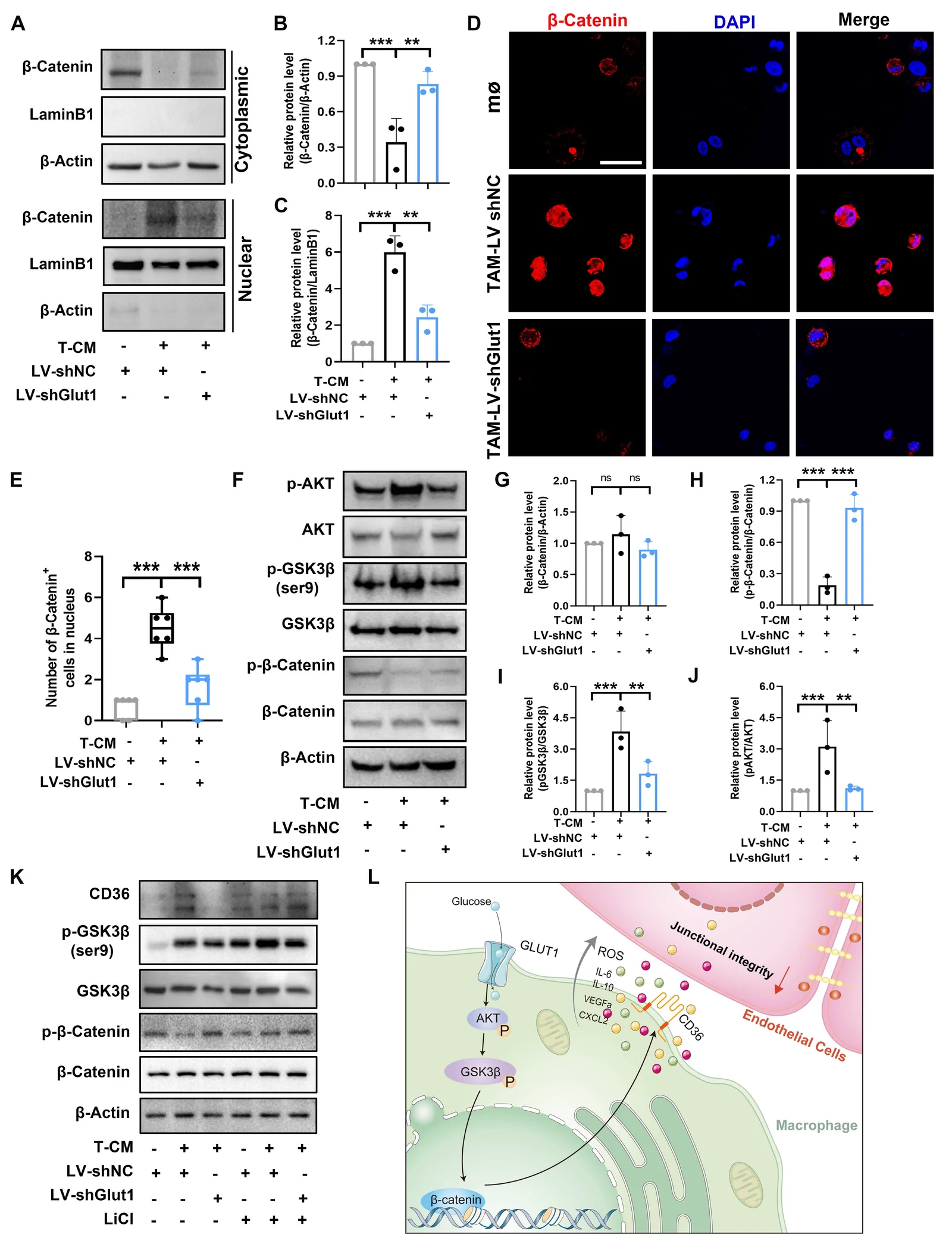
** Loss of Glut1 in macrophages regulates the GSK3β/β-catenin/CD36 signaling axis.** (A) The protein levels of β-catenin in the cytoplasm and nucleus were detected by western blot analysis after nuclear and cytoplasmic protein separation. (B) Quantitative analysis of the protein expression of cytoplasmic β-catenin. (C) Quantitative analysis of the protein expression of nuclear β-catenin. (D) Quantitative analysis of the number of cells with β-catenin^+^ nucleus (scale bars: 50 μm). (E) Representative IF images for co-staining of β-catenin and DAPI. (F) Western blot analysis for the expression of proteins in the AKT-GSK3β-βcatenin signaling axis. (G-J) Quantitative analysis of the expression of proteins in the AKT-GSK3β-βcatenin signaling axis. (G) Quantitative analysis of β-catenin/β-Actin. (H) Quantitative analysis of pβ-catenin/β-catenin. (I) Quantitative analysis of p-AKT/AKT. (J) Quantitative analysis of p-GSK3β/GSK3β. (K) Western blot analysis for the expression of proteins in the AKT-GSK3β-βcatenin signaling axis in the absence or presence of GSK-3β inhibitor LiCl. (L) Mechanism diagram of Glut1 in Macrophages influencing the endothelial integrity by regulating the AKT-GSK3β-βcatenin signaling axis. The data were presented as the mean ± SD and analyzed by one-way ANOVA. * *p*< 0.05, ** *p* < 0.01 and *** *p* < 0.001.

We subsequently explored whether GLUT1 was able to influence the expression of CD36 via regulating a specific signaling pathway. Previous studies have demonstrated that GLUT1 in tumor cells is capable of modulating the Akt/GSK-3β/β-catenin signaling axis 65. Crucially, β-catenin activation (either via LiCl stimulation in microglia or genetic stabilization in cancer cells) significantly upregulates CD36 expression, whereas inhibition of β-catenin conversely suppresses CD36 levels 66-68. In this regard, we investigated whether GLUT1 would affect the expression level of CD36 in TAMs through curbing β-catenin. Interestingly, it was illustrated that TCM treatment promoted the protein expression level of nuclear β-catenin in the macrophages, which could be rescued in the presence of LV-shGlut1 infection (Fig. 7A-7C). IF analysis further validated the elevated nuclear translocation of β-catenin upon the stimulation of TCM, which was diminished in response to the knockdown of GLUT1 (Fig. 7D and 7E). Further, TCM treatment led to the activation of the AKT/GSK-3β/β-catenin signaling pathway in macrophages, as demonstrated by the increased phosphorylation of AKT (Ser473) and GSK-3β (Ser9). Nevertheless, silence of GLUT1 inhibited the phosphorylation of these proteins at the specific sites and impeded the stabilization of β-catenin (Fig. 7F-7J). Additionally, we took advantage of LiCl that serves as an inhibitor of GSK3β (Glycogen Synthase Kinase 3 Beta) for further verification, and it was shown that the impacts of TCM on the expression level of β-catenin in the macrophages was prominently weakened following the treatment of LiCl (Fig. 7K). Meanwhile, LiCl treatment was prone to reduce the regulatory effects of macrophage supernatants on the mRNA expression levels of various endothelial adhesion-associated molecules including *Vcam1*, *Icam1*, and *Cd62e* (Fig. S5H-S5J). To sum up, our data implicate that macrophage-specific GLUT1 controls the expression of CD36 through regulating the AKT/GSK-3β/β-catenin signaling cascade. CD36, in return, exerts impacts on the metabolic functions of macrophages and modulates the secretion of multiple cytokines including VEGF, CXCL2, IL-6, and IL-10, ultimately influencing the biological behaviors of ECs (Fig. 7L).

### Preparation and characterization of M2 macrophage-targeted peptide functionalized nanoparticles

Our above findings uncovered that macrophage-specific deletion of Glut1 not only retarded tumor progression but also favorably remodeled the TIME, validating GLUT1 as a promising therapeutic target in TAMs. Nevertheless, the lack of effective approaches to specifically manipulate GLUT1 in macrophages by inhibitors has hindered its potential as a therapeutic target. To address this gap, we employed a nanotechnology-based targeting strategy 69, 70. PLGA nanoparticles, composed of polylactic acid (PLA) and polyglycolic acid (PGA), provide tunable properties including controlled degradation rates and drug release kinetics 71, 72. The capacity for sustained drug release is of great importance for cancer therapy as it enables to attenuate dosing frequency while enhance therapeutic efficacy 73. Moreover, their surface can be functionalized with targeting ligands that allow targeted delivery to specific cells via ligand-receptor interactions, thereby improving cellular uptake and intracellular drug release 74, 75. The M2 macrophage-targeting peptide (YEQDPWGVKWWY) was identified by Cieslewicz *et al*. via phage display screening (PhD C7 and PhD12 libraries) and selectively binds to murine CD45⁺F4/80⁺CD301⁺ TAMs 76. Consequently, subsequent studies have well-documented the capability of this peptide sequence to mediate precise drug delivery to M2 macrophages 77. WZB117, a well-characterized GLUT1 inhibitor 78, 79, was encapsulated into M2 macrophage-directed nanoparticles to achieve targeted delivery to pro-tumoral M2-like TAMs.

**Figure 8:**
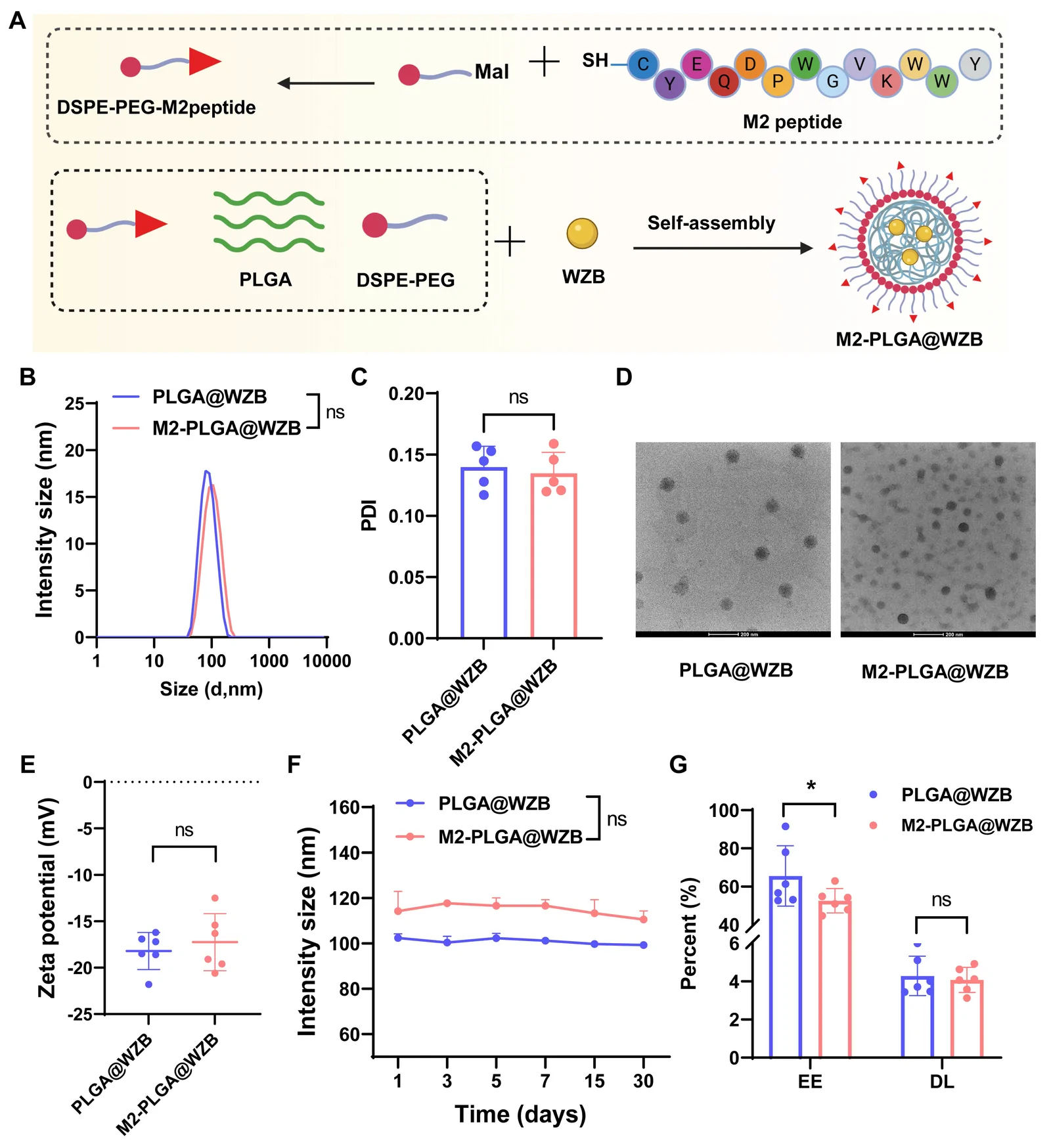
** Preparation and characterization of M2-PLGA@WZB117.** (A) Schematic diagram of the preparation of M2-PLGA@WZB117 nanoparticles. (B) Particle size distribution of PLGA@WZB117 and M2-PLGA@WZB117 nanoparticles. (C) The PDI of PLGA@WZB117 and M2-PLGA@WZB117 nanoparticles (n = 5). (D) Representative TEM image of PLGA@WZB117 and M2-PLGA@WZB117 nanoparticles. (E) The zeta potential of PLGA@WZB117 and M2-PLGA@WZB117 nanoparticles (n = 6). (F) Time-dependent changes in particle size of PLGA@WZB117 and M2-PLGA@WZB117 nanoparticles (n = 3). (G) EE and DL capacity of PLGA@WZB117 and M2-PLGA@WZB117 nanoparticles (n = 6).

As elucidated in Figure 8A, the M2 peptide-functionalized nanoparticles of WZB117 (M2-PLGA@WZB117) were prepared following a two-step procedure. Initially, to achieve high-efficiency conjugation via thiol-maleimide chemistry, a cysteine residue was introduced at the N-terminus of the M2 macrophage-targeting peptide (YEQDPWGVKWWY). The resulting modified peptide (CYEQDPWGVKWWY) was then covalently conjugated to DSPE-PEG-MAL to yield the functional ligand DSPE-PEG-M2. Subsequently, this ligand was co-assembled with PLGA polymer and the encapsulated drug WZB117 through a nanoprecipitation and self-assembly process, yielding core-shell structured nanoparticles with active targeting capability. Comprehensive physicochemical characterization validated the successful construction of the nanoparticles. More specifically, the narrow size distribution (Fig. 8B) and low polydispersity index (Fig. 8C) collectively highlighted the formation of a homogeneous and monodisperse nanoparticle population. Modification with the targeting ligand resulted in a slight increase in the mean hydrodynamic diameter from 88.18 nm (PLGA@WZB117) to 94.73 nm (M2-PLGA@WZB117), which was consistent with the presence of a surface-grafted PEG-peptide layer. The transmission electron microscopy (TEM) analysis unraveled the spherical morphology and excellent dispersion state without noticeable aggregation (Fig. 8D). Both formulations exhibited moderately negative zeta potentials of approximately -18 mV (Fig. 8E), indicative of good colloidal stability. This stability was further substantiated by the minimal changes in particle size after one month of storage at 4 °C (Fig. 8F). Importantly, the encapsulation efficiency and drug loading capacity were determined to be 65.56% ± 5.89% and 4.29% ± 0.38% for PLGA@WZB117, and 52.61% ± 2.37% and 4.08% ± 0.25% for M2-PLGA@WZB117, respectively (Fig. 8G). In summary, these comprehensive physicochemical characterizations unveil that the stable M2 peptide-targeted nanoparticles with well-defined structural characteristics were successfully generated, which were suitable for subsequent biological evaluation.

### M2-PLGA@WZB117 inhibits tumor progression and normalizes tumor blood vessels

In order to interrogate the therapeutic potential of the nanomedicines on tumors, we initially dissected the role of GLUT1 inhibitor WZB117 on the RAW264.7 macrophages, and it was observed that WZB117 profoundly attenuated glucose uptake of macrophages. Noteworthily, both PLGA@WZB117 and M2-PLGA@WZB117 were able to recapitulate the inhibitory effect on glucose uptake of macrophages, exhibiting the comparable efficacy to free WZB117 (Fig. S6A). Moreover, WZB117 treatment mitigated the secretion of multiple cytokines from TAMs including IL-6, IL-10, VEGF and CXCL2, mirroring the phenotypes observed in the GLUT1-knockdown models. In consistent with these phenomena, both PLGA@WZB117 and M2-PLGA@WZB117 displayed the robust suppression on the release of these cytokines, confirming their capabilities to functionally target GLUT1 in the macrophages (Fig. S6B-S6E).

**Figure 9:**
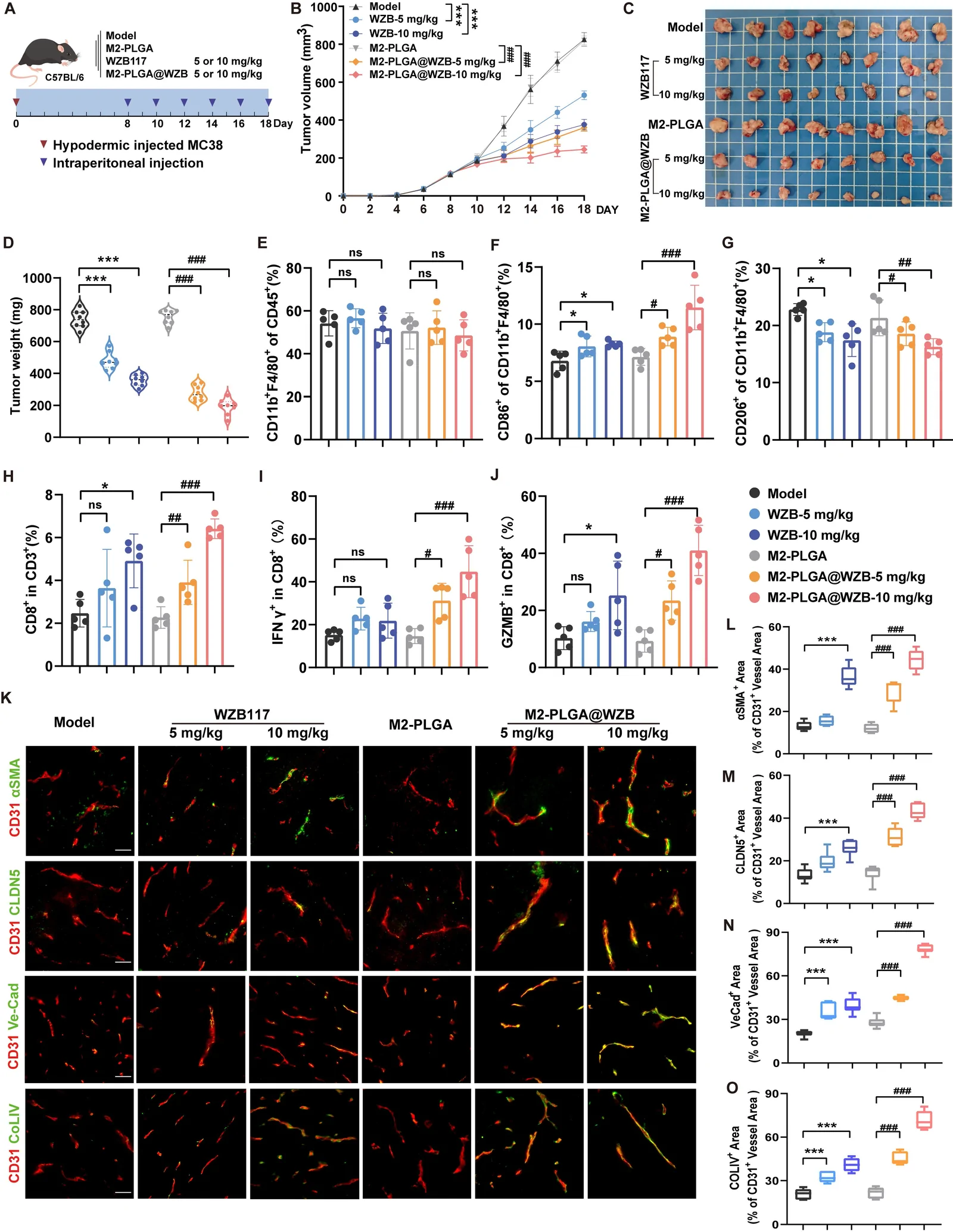
** M2-PLGA@WZB117 inhibits tumor growth and normalizes tumor blood vessels.** (A) Schematic diagram of mouse MC38 tumor model establishment and treatment regimens for WZB117 and M2-PLGA@WZB117. (B) The growth curves of mouse tumors upon different treatments. The data were presented as the mean ± SEM. (C) Photograph of mouse tumors in different groups (n = 8). (D) The weights of mouse tumors in different groups. (E-G) Flow cytometry analysis of macrophages in the mouse tumor tissues, including total macrophages (CD45^+^CD11b^+^F4/80^+^), M1-like macrophages (CD45^+^CD11b^+^F4/80^+^CD86^+^) and M2-like macrophages (CD45^+^CD11b^+^F4/80^+^CD206^+^). (H-J) Flow cytometry quantitative plots of total T cells (CD45^+^CD3^+^), CD8^+^T cells (CD45^+^CD3^+^CD8^+^), and CD4^+^T cells (CD45^+^CD3^+^CD4^+^) in the mouse tumor tissues (n = 5). (K) Representative IF images for co-staining of CD31 (red) with α-SMA, CLDN5, Ve-Cad and COL IV (green) in the tumor tissues (scale bars: 50 μm). (L-O) Quantification for the percentage of CD31^+^α-SMA^+^ area (L), CD31^+^CLDN5^+^ area (M), CD31^+^Ve-Cad^+^ area (N) and CD31^+^COL IV^+^ area (O) in the mouse tumor tissues. The data were presented as the mean ± SD and analyzed using one-way ANOVA. * *p*< 0.05, ** *p* < 0.01 and *** *p* < 0.001 and ^#^ *p*< 0.05, ^##^ *p* < 0.01 and ^###^ *p* < 0.001.

Aiming at exploiting the impacts of WZB117 on tumor progression *in vivo*, we thus established a subcutaneous MC38 tumor model in C57BL/6J mice. Drug treatment was initiated on day 8 following the inoculation of MC38 tumor cells, and then the tested drugs were administered into the C57BL/6J mice via intraperitoneal injection at doses of 5 or 10 mg/kg every two days (Fig. 9A). After six treatment cycles, the mice were euthanized by virtue of cervical dislocation, and the tumor tissues were harvested for the subsequent analysis. It was elucidated that treatment with WZB117 (5 mg/kg and 10 mg/kg) was able to significantly slow down the growth of tumors (Fig. 9 B). More interestingly, the macrophage-targeted nanomedicine for GLUT1 inhibitor (M2-PLGA@WZB117) at both 5 mg/kg and 10 mg/kg contributed to more conspicuous suppression in both tumor size as well as tumor weight compared to M2-PLGA vector (Fig. 9C and 9D). Meanwhile, histopathological analysis for the major organs including heart, liver, spleen, lung, and kidney tissues highlighted that there were no evident structural abnormalities or tissue damage across different treatment groups, suggesting the favorable biosafety of M2-PLGA@WZB117 (Fig. S7A). Additionally, although there were no prominent changes in terms of the infiltration of TAMs in the mice following the treatment with M2-PLGA@WZB117 at the doses of both 5 mg/kg and 10 mg/kg, the number of infiltrated CD86^+^ macrophages was significantly elevated while the number of infiltrated CD206^+^ macrophages was dramatically attenuated as compared to M2-PLGA vector (Fig. 9E-9G). Moreover, there were no pronounced alterations in light of the number of total CD3^+^ T cells in the tumor tissues (Fig. S7B). Nevertheless, M2-PLGA@WZB117 treatment not only resulted in a modest increase in total CD8⁺ T cells but also led to a dose-dependent elevation in the frequencies of IFN-γ⁺ and GZMB⁺CD8⁺ T cell subsets compared to the M2-PLGA vector group (Fig. 9H-9J, Fig. S7B). These findings implicate that M2-PLGA@WZB117 yields a robust enhancement in CD8⁺ T cell activation and their effector functions. Further, IF analysis demonstrated that the expression of α-SMA and Claudin-5 in the CD31^+^ blood vessels was significantly elevated in response to the treatment of 5 mg/kg or 10 mg/kg M2-PLGA@WZB117 (Fig. 9K-9M). Furthermore, compared with the control group, M2-PLGA@WZB117 treatment markedly improved VE-cadherin junction coverage and type IV collagen continuity (Fig. 9K, 9N and 9O). These results provide strong structural evidence that M2-PLGA@WZB117 effectively normalizes tumor blood vessels. In conclusion, all of these results clearly pinpoint that M2-PLGA@WZB117 as an effective and efficient GLUT1 inhibitor in macrophages plays a critical role in retarding the progression of tumors, suggesting that targeting macrophage GLUT1 may emerge as a promising therapeutic strategy for fighting against the progression of tumors.

## Discussion

Our study unveils a previously unrecognized role of macrophage-specific glucose metabolism in orchestrating the TIME. We demonstrate that genetic deficiency or pharmacological inhibition of GLUT1 in TAMs not only directly impairs tumor progression but also initiates a profound reprogramming of TME, leading to enhanced anti-tumor immunity and, most notably, the normalization of tumor vasculature. This triad of effects on the metabolic, immune, and vascular aspects of the TME positions GLUT1 in TAMs as a pivotal orchestrator of tumor progression and a therapeutic target of high promise.

The high expression of GLUT1 in TAMs of human and mouse tumors aligns with their known status as major glucose consumers in the TME 15, 18. Traditionally, the therapeutic inhibition of glycolysis in cancer has predominantly concentrated on cancer cells themselves 80, 81. Our study, however, establishes GLUT1 in TAMs as a distinct and promising metabolic checkpoint. The depletion of GLUT1 in macrophages shifts the phenotypes of TAMs from a pro-angiogenic (CD206⁺) state toward a pro-inflammatory (CD86⁺) state, thus reprogramming them toward a tumor-suppressive phenotype. This *Glut1*-mediated mechanism operates broadly, as evidenced by significant tumor growth inhibition in multiple syngeneic models, including colon and lung cancers. Targeting GLUT1 in TAMs represents a promising therapeutic strategy for retarding the progression of tumors.

A pivotal and translationally relevant finding is the induction of tumor vascular normalization following GLUT1 deletion in TAMs. Tumor development requires a supportive microenvironment characterized by neoangiogenesis and chronic immune filtration 82. Abnormal tumor vasculature poses a major barrier to immunotherapy by compromising drug delivery and effector T cell infiltration 11, 83-86. Consequently, reprogramming the TIME through vascular normalization represents a promising therapeutic strategy. Our study indicates that GLUT1-reprogrammed TAMs initiate a self-reinforcing, anti-tumoural circuit within the TME. GLUT1 inhibition reprograms TAMs via the GLUT1/β-catenin/CD36 signaling axis, thereby altering the secretion of multiple cytokines including CXCL2, VEGF, IL-10, and IL-6 to indirectly influence the tumor vascular structure and function. The normalized vasculature, in turn, facilitates the enhanced infiltration and function of anti-tumor immune cells, as evidenced by the elevated frequencies of IFN-γ⁺ and GZMB⁺CD8⁺ T cells. The mechanistic insight establishes a clear link between TAM glucose metabolism and vascular remodeling. While TAM glycolysis has been broadly implicated in tumor progression and altered vascular function, the precise pathway linking macrophage glucose uptake to angiogenesis through the GLUT1/β-catenin/CD36 signaling axis has not been defined. This paves the way for the novel niche highlighted in our study. Our findings collectively demonstrate that metabolic reprogramming of TAMs remodels the TME through vascular normalization, ultimately restraining tumor progression.

In this study, we identified CD36 as a key downstream effector of the GSK3β/β-catenin signaling axis in GLUT1-deficient TAMs, indicating that loss of GLUT1 primarily modulates TAM function through this signaling pathway. Indeed, CD36 is a well-established fatty acid translocase, and CD36-mediated lipid uptake has been reported to promote TAM differentiation and support tumor growth 87. This raises an important question of whether inhibition of glucose metabolism could trigger a compensatory shift toward fatty acid utilization, a common survival strategy in nutrient-deprived cells 17, 88. Although our current study did not directly investigate such metabolic adaptation, it represents a significant area for future research. Exploring whether GLUT1-deficient TAMs switch their metabolic reliance to fatty acids may provide deeper insights into TAM metabolic plasticity, functional polarization, and their interactions with tumor cells and immune cells in the TME.

Despite elucidating that repression of GLUT1 in TAMs influences cytokine release via CD36 and impacts EC behavior, there are still several limitations. More specifically, we have not been able to track the interactions between macrophages and TECs in a real-time manner in the tumor-bearing mice, nor fully demonstrate the specific molecular pathways governing their crosstalk. In the subsequent studies, gain- and loss-of-function experiments may further help to validate and explore the potential interactions between TAMs and TECs. In addition, it was observed that possible metabolic competition might exist between these two cell types. Further studies to take advantage of metabolomic profiling, metabolite flux analysis, and alternative biochemical approaches may be required to characterize the alterations in metabolic states as well as to clarify the modes and signaling pathways governing the interactions between TAMs and TECs.

Although TAMs exhibit high metabolic and functional heterogeneity, our data show that GLUT1 deficiency shifts their polarization from M2-like to M1-like phenotype. It indicates that the observed anti-tumor efficacy predominantly stems from modulating the immunosuppressive M2 subset of macrophages, providing a strong rationale for developing the M2-targeted M2-PLGA@WZB nanoparticle platform. Of note, the current study was primarily conducted within the conventional M1/M2 polarization framework, whereas the functional diversity of TAMs within the TME is likely far more complicated. Future studies integrating single-cell transcriptomics, spatial profiling, and subset-specific genetic strategies will be valuable for further dissecting the context-dependent roles of distinct TAM populations.

While the Csf1r-Cre model provided robust evidence for the specific role of macrophage GLUT1 in our study, future cross-validation using complementary genetic models (such as inducible Cre lines or alternative lineage-specific drivers) would help to rule out potential strain-specific biases and further corroborate the specificity of macrophage-intrinsic GLUT1 deletion. Beyond ensuring model specificity, it is also crucial to consider the broader cellular context. While our study primarily elucidated the role of GLUT1 in TAMs and its subsequent impacts on tumor progression, it is important to note that GLUT1 is widely expressed across diverse cell types within the TME. For instance, tumor cells exploit GLUT1-driven glycolysis to evade T cell killing, while targeting GLUT1 can enhance T cell-mediated killing 89. GLUT1 overexpression in CAR-T cells promotes metabolic reprogramming and cytotoxic potency 90. Consequently, a comprehensive evaluation of the multifaceted roles of GLUT1 across the multicellular landscape of the TME remains essential for a holistic understanding of tumor pathogenesis. Notably, TAMs tend to interact with multiple cell types within the TME, including TECs, tumor cells, and CD8⁺ T cells. While our study primarily focused on the TAMs-TECs interactions, GLUT1 deficiency in TAMs may also exert indirect effects on other compartments via altered cytokine and metabolite secretion. These observations highlight potential avenues for future research and open the door to further investigations into how TAM metabolic reprogramming reshapes the broader TME network.

Furthermore, the multicellular complexity of the TME necessitates cautious evaluation of potential off-target or systemic effects of GLUT1 inhibition. Although our M2-PLGA@WZB drug delivery platform achieves preferential targeting toward M2-like TAMs, the bystander impacts of WZB117 on other TME components, primarily tumor cells and Ecs, cannot be entirely dismissed. Crucially, tumor cells are critically dependent on GLUT1-mediated glycolysis to sustain the Warburg phenotype 91, whereas TECs rely heavily on glycolytic metabolism during active angiogenesis 92. Therefore, localized leakage or nonspecific uptake of WZB117 may indirectly contribute to anti-tumor efficacy by suppressing tumor cell metabolism or impairing vascular sprouting. Nevertheless, these insights should be interpreted within the context of a preliminary proof-of-concept study. Several translational challenges remain to be addressed before clinical translation can be realized, including the optimization of targeting specificity, scalable large-scale manufacturing, long term biosafety assessments, and comprehensive pharmacokinetic/pharmacodynamic characterization.

Despite these limitations, our findings provide novel perspectives on macrophage-targeted metabolic therapies for cancer treatment. We developed M2-PLGA@WZB117, a macrophage-targeted nanoparticle that delivers the GLUT1 inhibitor WZB117 specifically to M2-like macrophages. The significant anti-tumor efficacy of this formulation, coupled with its ability to recapitulate the vascular normalization phenotype observed in genetic models, validates GLUT1 as a druggable target in TAMs. This targeted strategy circumvents the potential toxicity of systemic GLUT1 inhibition while maximizes therapeutic impacts through targeted delivery to the relevant cellular compartment within the TME. Our work thus provides a compelling rationale for developing macrophage-targeted metabolic therapies. Also, targeting GLUT1 in TAMs presents a unique advantage: it simultaneously remodels the physical structure of the TME (vasculature) and enhances the anti-tumor immune landscape. This dual action could potentially overcome resistance to existing therapies. A key future objective is to explore the potential synergy with PD-1/PD-L1 blockade, which is imperative for advancing this targeted strategy into novel combination therapies for clinical application.

Taken together, our study provides novel insights for reprogramming TAMs and elucidating the interactions between TAMs and tumor blood vessels. Depletion of GLUT1 in macrophages results in the restricted tumor growth, altered polarization of TAMs toward an antitumor phenotype, enhanced antitumor T-cell immunity, and induced tumor vascular normalization. Targeting GLUT1 or manipulating its downstream signaling pathways (such as GLUT1/β-catenin/CD36 signaling cascade) may represent a novel therapeutic strategy to manipulate the functions of TAMs and improve the effectiveness of clinical immunotherapies.

## Supplementary Material

Supplementary figures and tables.
