# Supplementary material for: Deficiency of GLUT1 in macrophages reprograms tumor-associated macrophages to normalize tumor vasculature and retards the progression of tumors
Source: Int J Biol Sci. 2026 Jul 30;22(13):7127–50. doi: 10.7150/ijbs.131425 (PMC13540664; doi:10.7150/ijbs.131425)
Supplement: Supplementary file 1 [file ijbsv22p7127s1.pdf]

Supporting Information

Supplementary Table

Table S1. Oligo sequences of Glut1 shRNA

Name	Oligo Sequence
Glut1-i1-F	gatccgTGAGGAGTTCTACAATCAAACctcgagGTTTGATTGTAGA ACTCCTCAtttttt
Glut1-i1-R	aattaaaaaaTGAGGAGTTCTACAATCAAACctcgagGTTTGATTGT AGAACTCCTCAcg

Table S2. Sequences of primers used for RT–qPCR in this study

Primer	Sequences (5' to 3')	Species
Glut1-F	GCCAGGACCCACTTCAAAGA	Mus
Glut1-R	TTGGCTCCGGTATCGTCAAC	Mus
Actb-F	TGTTACCAACTGGGACGACA	Mus
Actb-R	GGGGTGTTGAAGGTCTCAAA	Mus
Arg1-F	TTTAGGGTTACGGCCGGTG	Mus
Arg1-R	TCCTCGAGGCTGTCCTTTTG	Mus
Nos2-F	CAGATCGAGCCCTGGAAGAC	Mus
Nos2-R	CTGGTCCATGCAGACAACCT	Mus
Cd36-F	ATGGGCTGTGATCGGAACTG	Mus
Cd36-R	TTTGCCACGTCATCTGGGTTT	Mus
VCAM1-Hu-Fwd	CATGACCTGTTCCAGCGAGG	Humo

VCAM1-Hu-Rvs	CATTCACGAGGCCACCACTC	Humo
ICAM1-Hu-Fwd	AGCAGACTCCAATGTGCCAG	Humo
ICAM1-Hu-Rvs	GACAGTCACTGATTCCCCGA	Humo
CD62E-Hu-Fwd	CAGCAAAGGTACACACACCTG	Humo
CD62E-Hu-Rvs	CAGACCCACACATTGTTGACTT	Humo

Supplementary Figures:

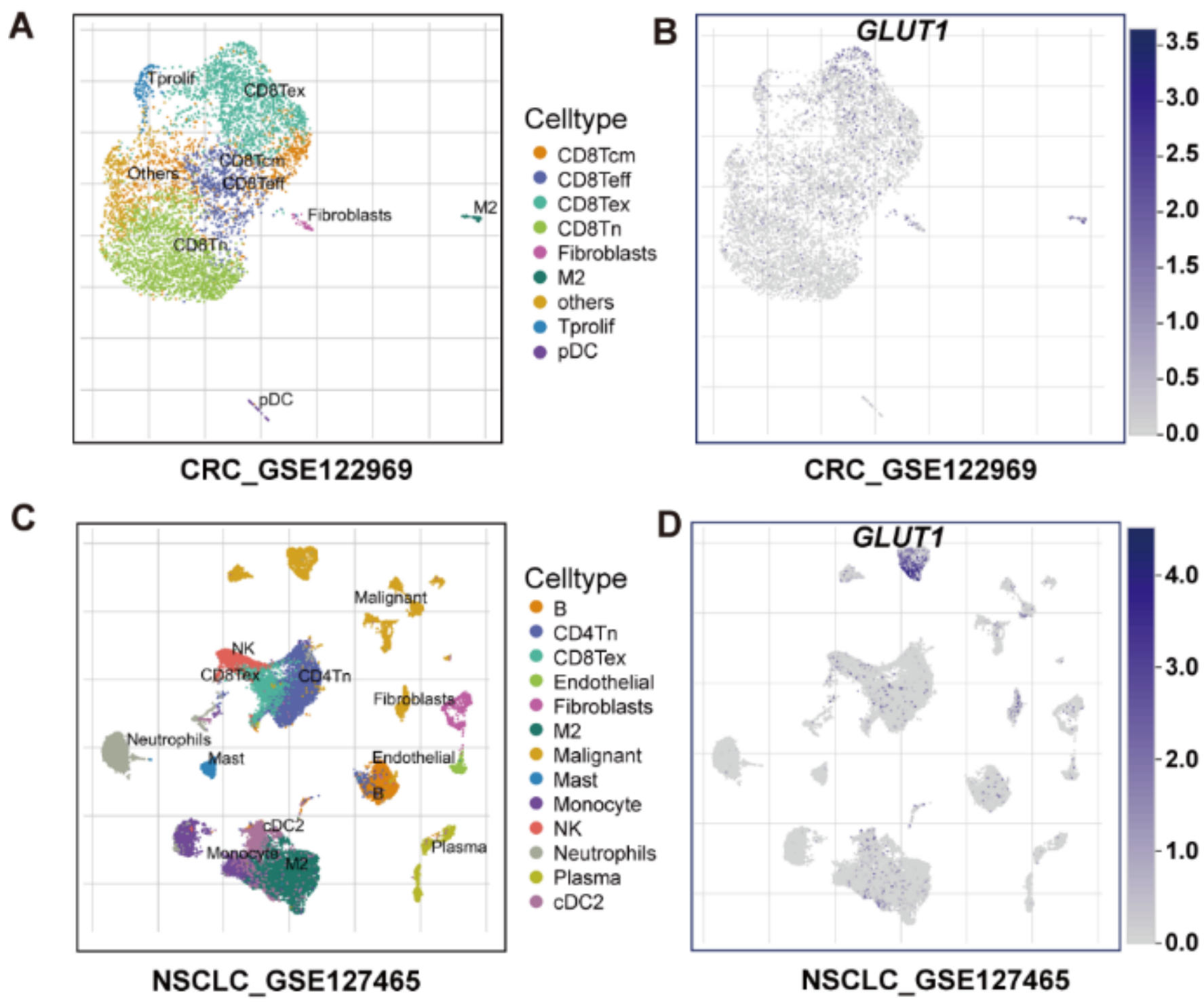

Figure S1. Expression of *Glut1* gene in different types of cells in the colorectal cancer (CRC) and non-small cell lung cancer (NSCLC). (A) Cell clustering analysis of scRNA-seq in the CRC_GSE122969_mouse_aPD1aTIM3 dataset. (B) The expression of *Glut1* gene in different types of cells in the CRC_GSE122969_mouse_aPD1aTIM3 dataset. (C) Cell clustering analysis of scRNA-seq in the NSCLC_GSE127465

dataset. (D) The expression of *GLUT1* gene in different types of cells in the NSCLC_GSE127465 dataset.

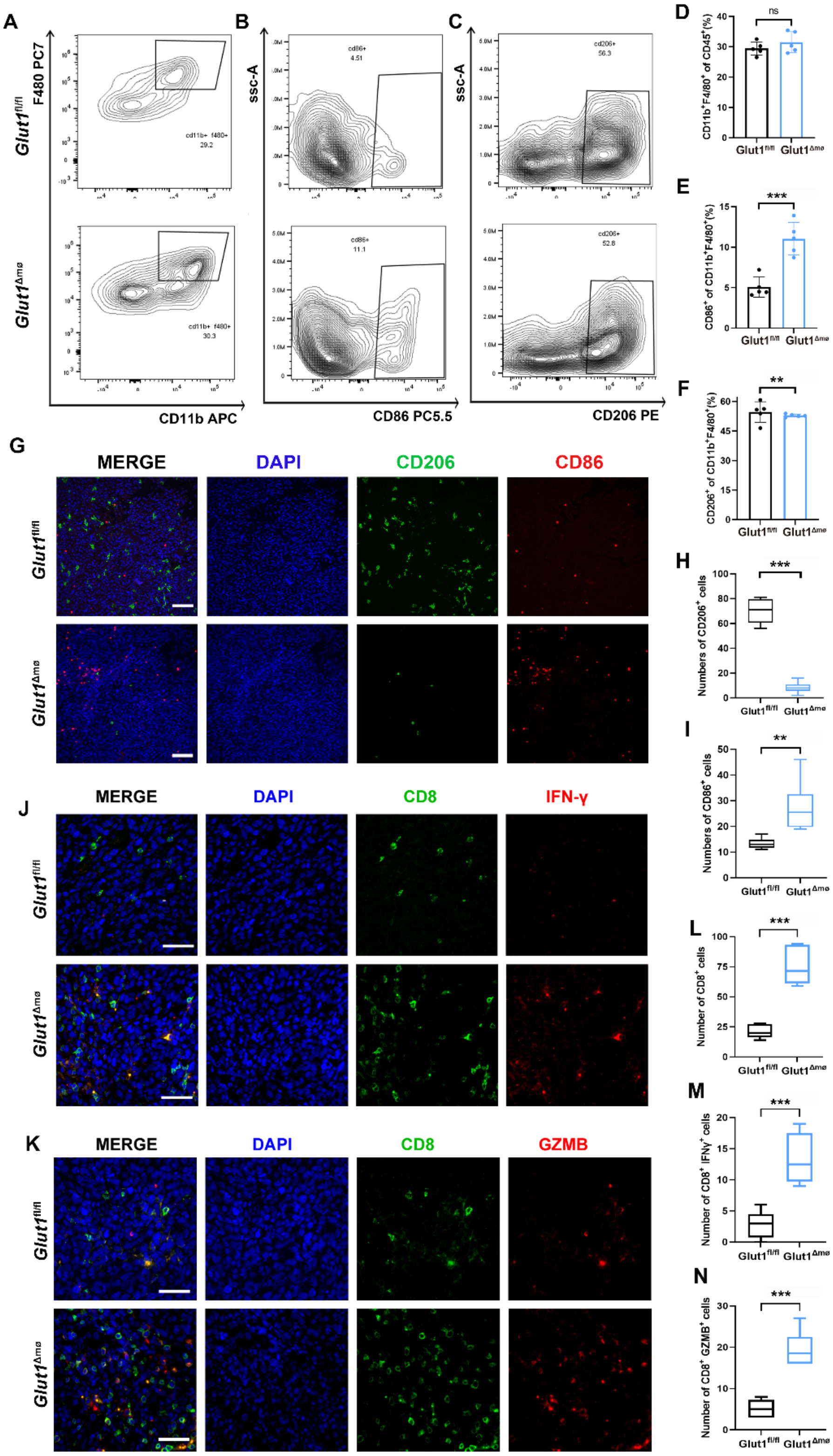

Figure S2. Macrophage-specific deletion of Glut1 strengthens the tumor immune microenvironment in the LLC tumor-bearing mice. (A-C) Flow cytometry analysis of macrophages in the LLC tumors including total macrophages ($CD45^+CD11b^+F4/80^+$), M1 macrophages ($CD45^+CD11b^+F4/80^+CD86^+$), M2 macrophages ($CD45^+CD11b^+F4/80^+CD206^+$) in the LLC tumor tissues. (D-F) Quantification for the flow cytometry analysis of total macrophages ($CD45^+CD11b^+F4/80^+$), M1 macrophages ($CD45^+CD11b^+F4/80^+CD86^+$), M2 macrophages ($CD45^+CD11b^+F4/80^+CD206^+$) in the LLC tumor tissues (n = 5). (G) Representative immunofluorescence images for co-staining of CD206 (green), CD86 (red) and DAPI (blue) in the LLC tumor-bearing mice (scale bars: 100 μm). (H and I) Quantification for the number of $CD206^+$ or $CD86^+$ cells in the visual field in the LLC tumor-bearing mice. (J) Representative immunofluorescence images for co-staining of $CD8^+$ (green) and IFNγ (red) in the LLC tumor tissues (scale bars: 50 μm). (K) Representative immunofluorescence images for co-staining of $CD8^+$ (green) and GZMB (red) in the LLC tumor tissues (scale bars: 50 μm). (L) Quantification of the number of $CD8^+$ cells in the LLC tumor tissues in the visual field. (M) Quantification of the number of $CD8^+IFN\gamma^+$ cells in the LLC tumor tissues in the visual field. (N) Quantification of the number of $CD8^+GZMB^+$ cells in the LLC tumor tissues in the visual field. The data were presented as the mean ± SD and analyzed by student's *t* - test. * $p < 0.05$, ** $p < 0.01$ and *** $p < 0.001$.

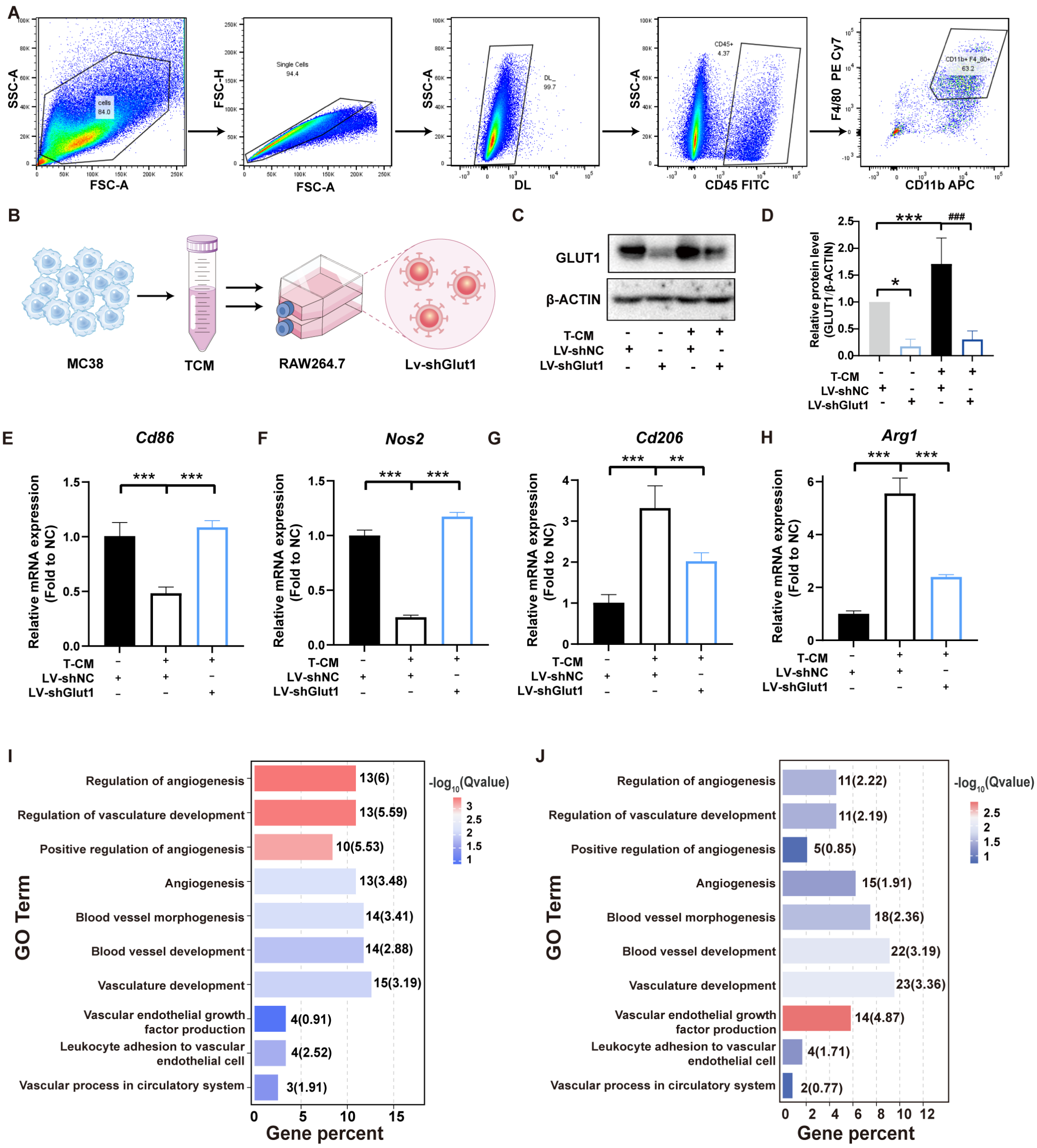

Figure S3. Transcriptome sequencing of TAMs both *in vitro* and *in vivo*.

(A) Gating strategy for the flow cytometry sorting of the primary TAMs derived from the MC38 tumor-bearing mice. (B) Schematic diagram for constructing TAMs with knockdown of GLUT1 by virtue of RAW264.7 cells. (C) Western blot bands of GLUT1 protein expression in the RAW264.7 cells. (D) Quantification of protein expression of GLUT1 upon different treatments. (E-H) The mRNA expression levels of *Cd86, Nos2, Cd206, Arg1* in the RAW264.7 cells after the

intervention of tumor cell supernatants. (I) GO enrichment analysis diagram for transcriptome sequencing of TAMs derived from mice. (J) GO enrichment analysis diagram for transcriptome sequencing in the RAW264.7 cells. The data were presented as the mean ± SD and analyzed by student's t - test and one-way ANOVA. * p< 0.05, ** p < 0.01 and *** p < 0.001.

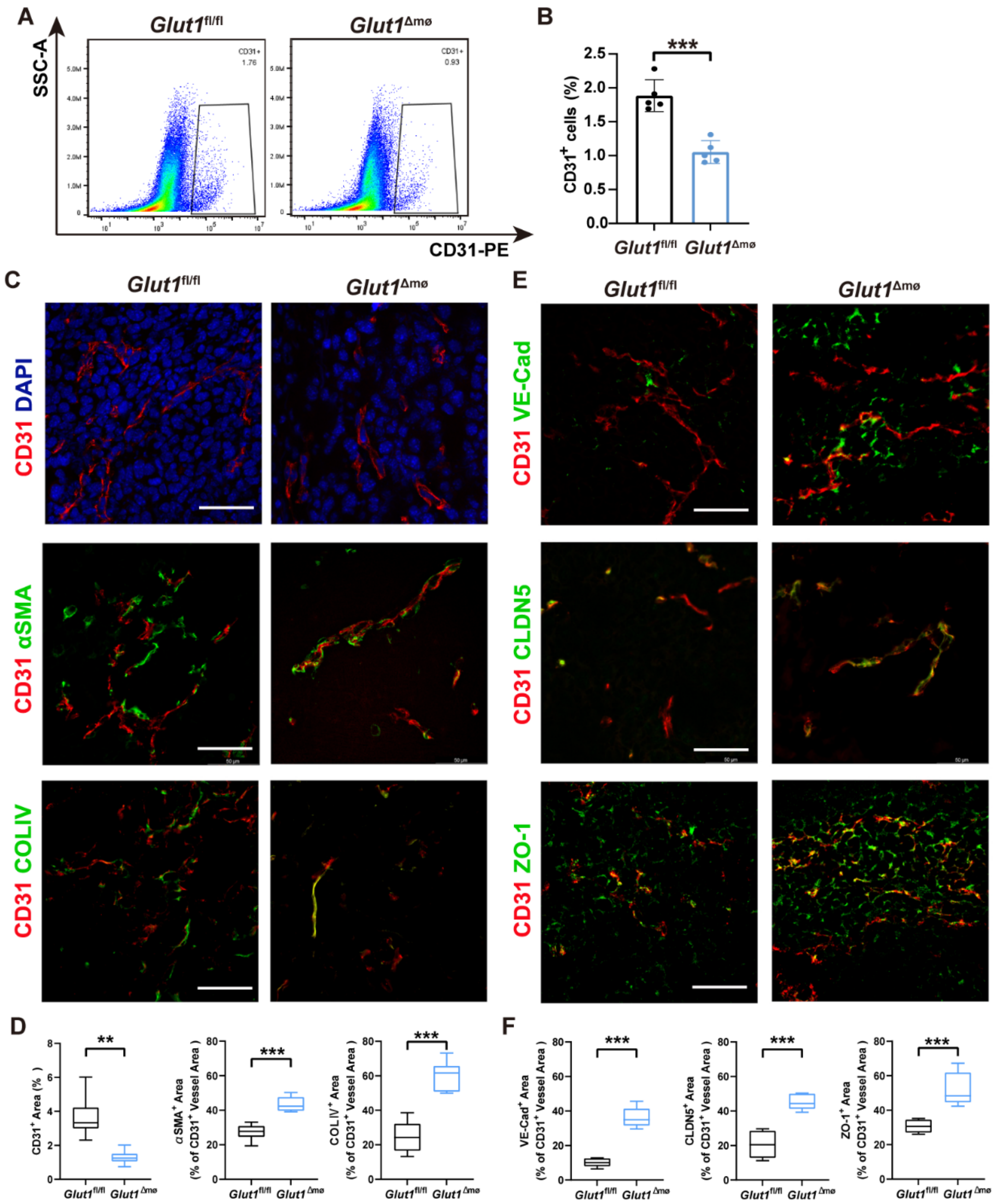

Figure S4. Macrophage-specific deletion of Glut1 normalizes tumor blood vessels in the LLC tumor-bearing mice. (A) Flow cytometry analysis for the percentage of ECs ($CD45^-CD31^+$) in the LLC tumors. (B) Representative flow cytometric analysis of ECs in the LLC tumors (n = 5). (C) Representative immunofluorescence images for co-staining of CD31 (red) with DAPI (blue), α-SMA (green) or COL IV (green) in the LLC tumors (scale bars: 50 μm). (D) Quantification for the number of blood vessels, as well as the percentage of $CD31^+$α-SMA^+ area or $CD31^+$COL IV $^+$ area in the LLC tumors (n = 6). (E) Representative immunofluorescence images for co-staining of CD31 (red) with VE-Cad (green), CLDN5 (green) or ZO-1 (green) in the LLC tumors (scale bars: 50 μm). (F) Quantification for the number of blood vessels, as well as the percentage of $CD31^+$VE-Cad^+ area, $CD31^+$CLDN5 $^+$ area or $CD31^+$ZO-1^+ area in the LLC tumors (n = 6). The data were presented as the mean ± SD and analyzed by student's *t* - test. * $p< 0.05$, ** $p < 0.01$ and *** $p < 0.001$.

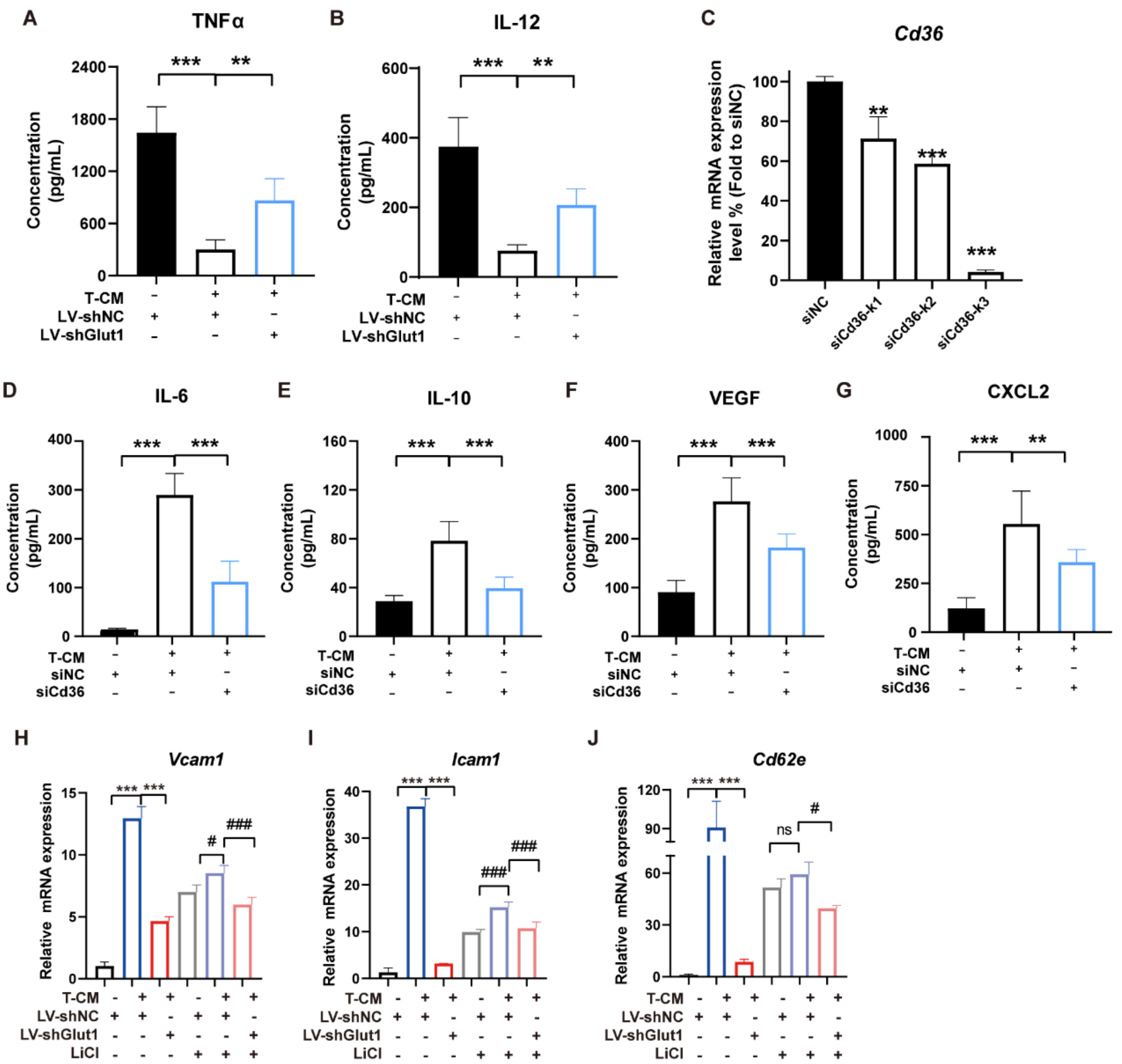

Figure S5. Macrophage GLUT1 regulates the Akt/GSK-3β/β-catenin-CD36 signaling axis. The levels of TNF-α (A) and IL-12 (B) in the supernatants of RAW264.7 macrophages were measured by ELISA. (C) After transfection of RAW264.7 cells with three types of siCd36, the mRNA level of *CD36* was examined. siCd36-k3 with the highest knockdown efficiency was selected for subsequent experiments. The levels of IL-6 (D), IL-10 (E), VEGF (F) and CXCL2 (G) in the supernatants of macrophages were measured by ELISA. (H) The mRNA expression levels of *Vcam1* upon different treatments were examined. (I) The mRNA expression levels of *Icam1* upon different treatments.

(J) The mRNA expression levels of *Cd62e* upon different treatments. The data were presented as the mean ± SD and analyzed by two-way ANOVA. * $p < 0.05$, ** $p < 0.01$ and *** $p < 0.001$; # $p < 0.05$, ## $p < 0.01$ and ### $p < 0.001$.

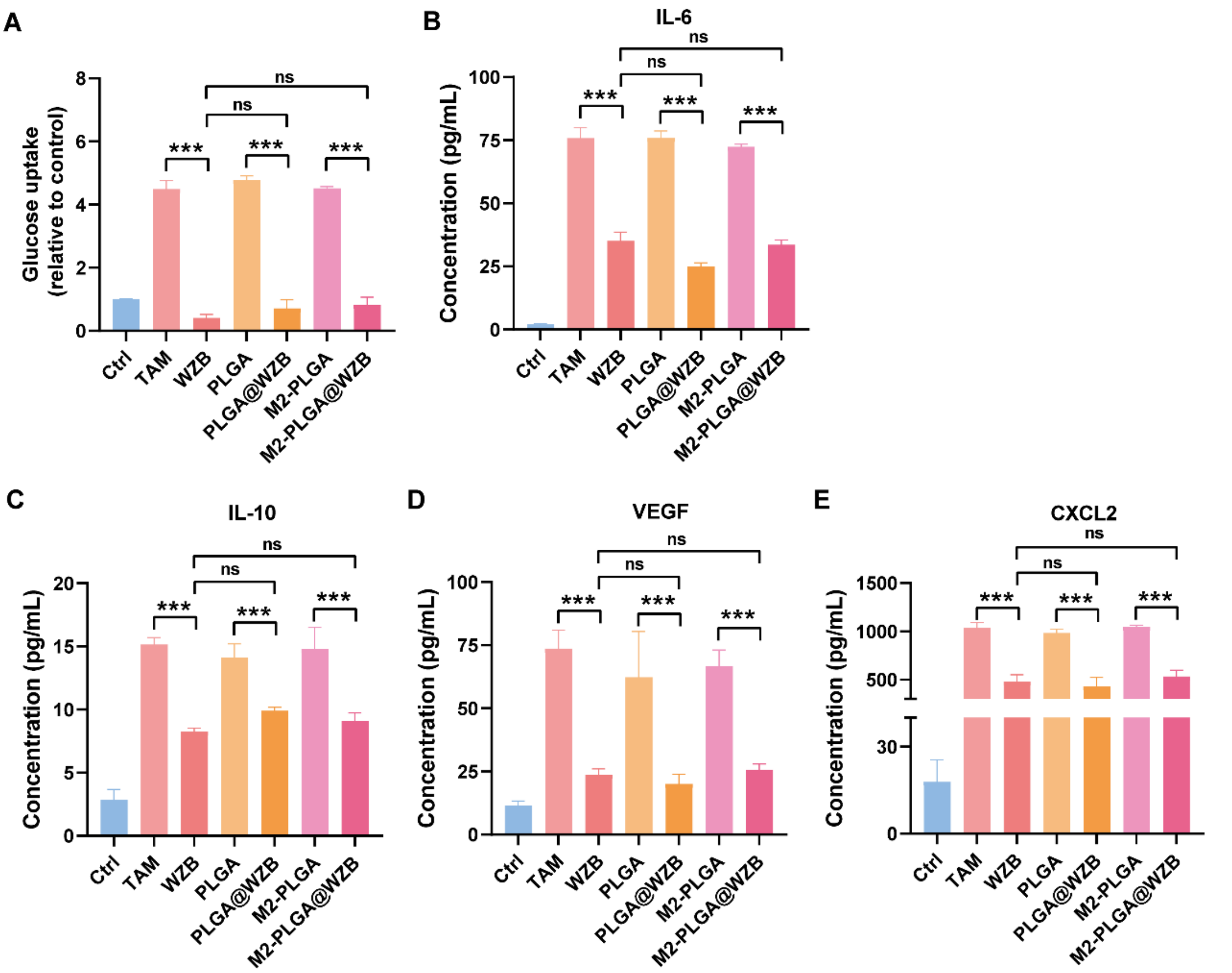

Figure S6. M2-PLGA@WZB117 inhibits glucose uptake and mitigates the secretion of critical cytokines and chemokines in macrophages. (A) The RAW264.7 macrophages were treated with different agents, and the glucose concentrations in the cell supernatants were measured to evaluate the glucose uptake capability. The levels of IL-6 (B), IL-10 (C), VEGF (D) and CXCL2 (E) in the supernatants of RAW264.7 macrophages treated with different formulations. The data were presented as the mean ± SD and analyzed by student's *t* - test

and one-way ANOVA. * p< 0.05, ** p < 0.01 and *** p < 0.001.

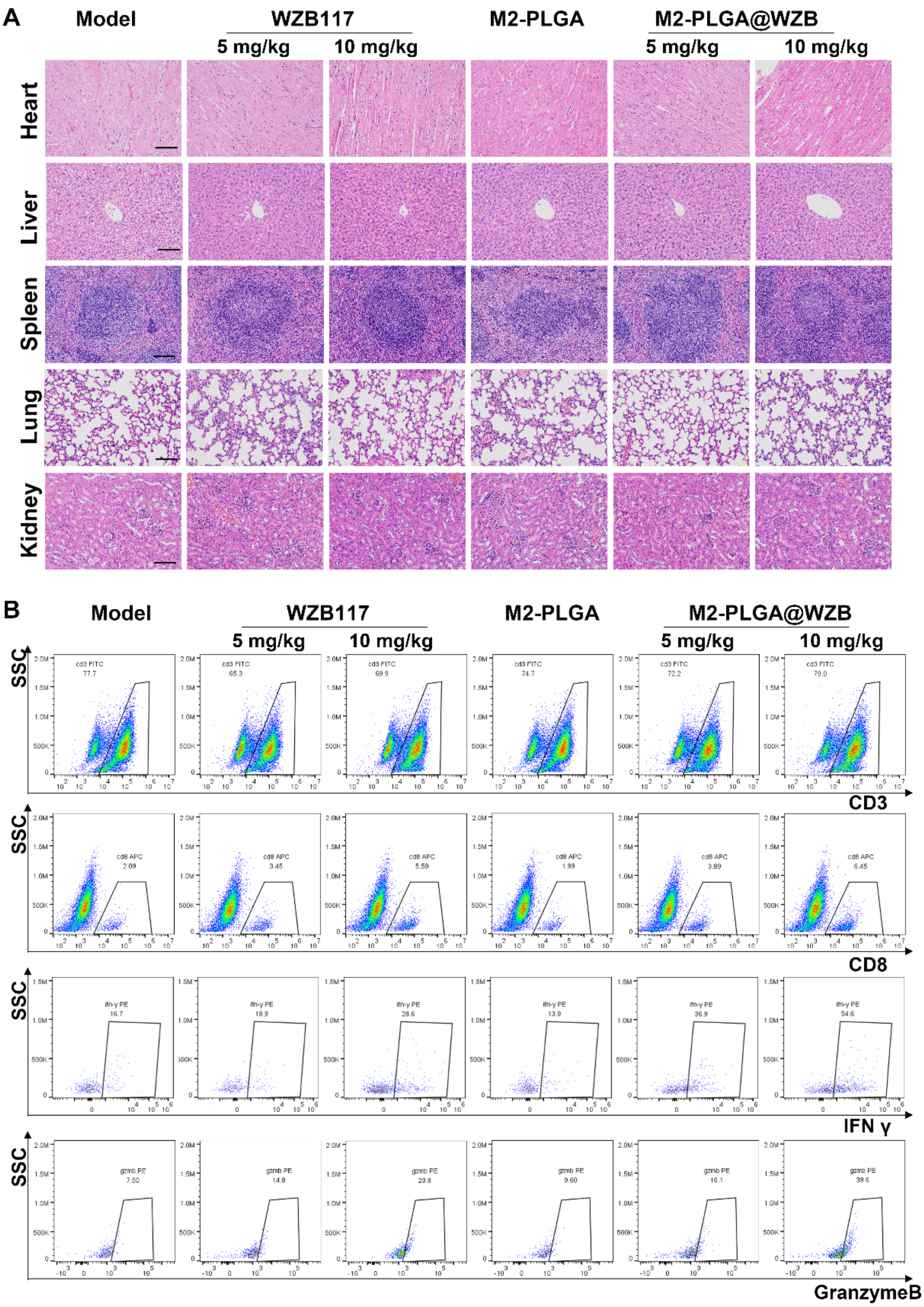

Figure S7. M2-PLGA@WZB117 inhibits tumor progression and normalizes

tumor blood vessels. (A) Representative H&E staining images of heart, liver, spleen, lung and kidney tissues following different treatments. (B) Flow cytometry analysis of total T cells ($CD3^{+}$), $CD8^{+}$T cells ($CD3^{+}CD8^{+}$), $IFN\gamma^{+}$T cells ($CD3^{+}CD8^{+}IFN\gamma^{+}$), and $GZMB^{+}$T cells ($CD3^{+}CD8^{+}GZMB^{+}$) in the MC38 tumor tissues (n = 5).

List of abbreviations

ACC	Adrenocortical carcinoma
BMDMs	bone marrow-derived macrophages
BRCA	Breast invasive carcinoma
CD36	Cluster of Differentiation 36
CESC	Cervical squamous cell carcinoma and endocervical adenocarcinoma
CLDN5	Claudin-5
COAD	colon adenocarcinoma
COL IV	collagen IV
CRC	colorectal cancer
DEG	differentially expressed genes
FACS	fluorescence activated cell sorting
GBM	Glioblastoma multiforme
GLUT1	glucose transporter protein 1
GO	Gene Ontology
GTEx	The Genotype-Tissue Expression
GZMB	Granzyme B
HNSC	Head and neck squamous cell carcinoma
HUVEC	Human Umbilical Vein Endothelial Cells
H&E	Hematoxylin-eosin
IF	immunofluorescence
IFN-γ	interferon-γ
KO	knockout
KIRP	kidney renal papillary cell carcinoma
LCA	lung cancer
LLC	lewis lung carcinomas cells

LUAD	Lung adenocarcinoma
LUSC	lung squamous cell carcinoma
OV	Ovarian serous cystadenocarcinoma
PAAD	pancreatic adenocarcinoma
READ	Rectum adenocarcinoma
RNA-seq	RNA sequencing
RT-qPCR	Quantitative reverse transcription PCR
scRNA-seq	single-cell RNA sequencing
SMCs	smooth muscle cells
STAD	Stomach adenocarcinoma
TAMs	tumor-associated macrophages
TAMs-CM	TAMs-conditioned media
TCGA	The Cancer Genome Atlas
TCM	tumor-conditioned medium
TECs	tumor-associated endothelial cells
TEM	transmission electron microscopy
TGCT	Testicular Germ Cell Tumors
TJs	tight junctions
TIME	tumor immune microenvironment
TISCH	Tumor Immune Single-Cell Hub
TME	tumor microenvironment
UCEC	uterine corpus endometrial carcinoma
USC	Uterine Carcinosarcoma
VE-Cad	VE-Cadherin
